# Phylogeography and cryptic species structure of a locally adapted parasite in New Zealand

**DOI:** 10.1111/mec.16570

**Published:** 2022-07-04

**Authors:** Frida Feijen, Natalia Zajac, Christoph Vorburger, Isabel Blasco‐Costa, Jukka Jokela

**Affiliations:** ^1^ Eawag, Swiss Federal Institute of Aquatic Science and Technology Dübendorf Switzerland; ^2^ Department of Environmental Systems Sciences ETH‐Zurich, Institute of Integrative Biology Zürich Switzerland; ^3^ Functional Genomics Center Zürich ETH Zürich/University of Zürich Zürich Switzerland; ^4^ Natural History Museum of Geneva Geneva 6 Switzerland; ^5^ Department of Arctic and Marine Biology UiT The Arctic University of Norway Tromsø Norway

**Keywords:** phylogeography, population genetics, New Zealand, species delimitation, mitonuclear discordance

## Abstract

The phylogeographic patterns of many taxa on New Zealand's South Island are characterized by disjunct distributions that have been attributed to Pleistocene climatic cycles and the formation of the Southern Alps. Pleistocene glaciation has been implicated in shaping the contemporary genetic differentiation between populations of the aquatic snail *Potamopyrgus antipodarum*. We investigated whether similar phylogeographic patterns exist for the snail's locally adapted trematode parasite, *Atriophallophorus winterbourni*. We found evidence for a barrier to gene‐flow in sympatry between cryptic, but ecologically divergent species. When focusing on the most common of these species, disjunct geographic distributions are found for mitochondrial lineages that diverged during the Pleistocene. The boundary between these distributions is found in the central part of the South Island and is reinforced by low cross‐alpine migration. Further support for a vicariant origin of the phylogeographic pattern was found when assessing nuclear multilocus SNP data. Nuclear and mitochondrial population differentiation was concordant in pattern, except for populations in a potential secondary contact zone. Additionally, we found larger than expected differentiation between nuclear‐ and mitochondrial‐based empirical Bayes *F*_ST_ estimates (global *F*_ST_: 0.02 vs. 0.39 for nuclear and mitochondrial data, respectively). Population subdivision is theoretically expected to be stronger for mitochondrial genomes due to a smaller effective population size, but the strong difference here, together with mitonuclear discordance in a putative contact zone, is potentially indicative of divergent gene flow of nuclear and mitochondrial genomes.

## 1 INTRODUCTION

Typical phylogeographic patterns for the biota on New Zealand's South Island include cross‐alpine and North–South splits in the distribution of divergent lineages (Trewick & Wallis, 2001; Wallis et al., 2016; Wallis & Trewick, 2009). The repeated occurrence of a distributional break, or gap, around the central area of the island is often referred to as the beech gap due to the absence of *Nothofagus* trees (Trewick & Wallis, 2001; Wallis et al., 2016). This gap has been attributed to processes such as the formation of the Southern Alps, Pleistocene glacial cycles or, for more ancient distribution patterns above species level, displacement of land masses along the Alpine Fault (Fleming, 1975; Haase et al., 2007; Wallis & Trewick, 2009; Wardle, 1963). Although some controversy exists, increasing evidence from divergence dating indicates that the Pleistocene glacial cycles and the formation of the Southern Alps are the most likely drivers of genetic differentiation on the South Island of New Zealand (Trewick & Wallis, 2001; Wallis et al., 2016; Wallis & Trewick, 2009).

**FIGURE 1:**
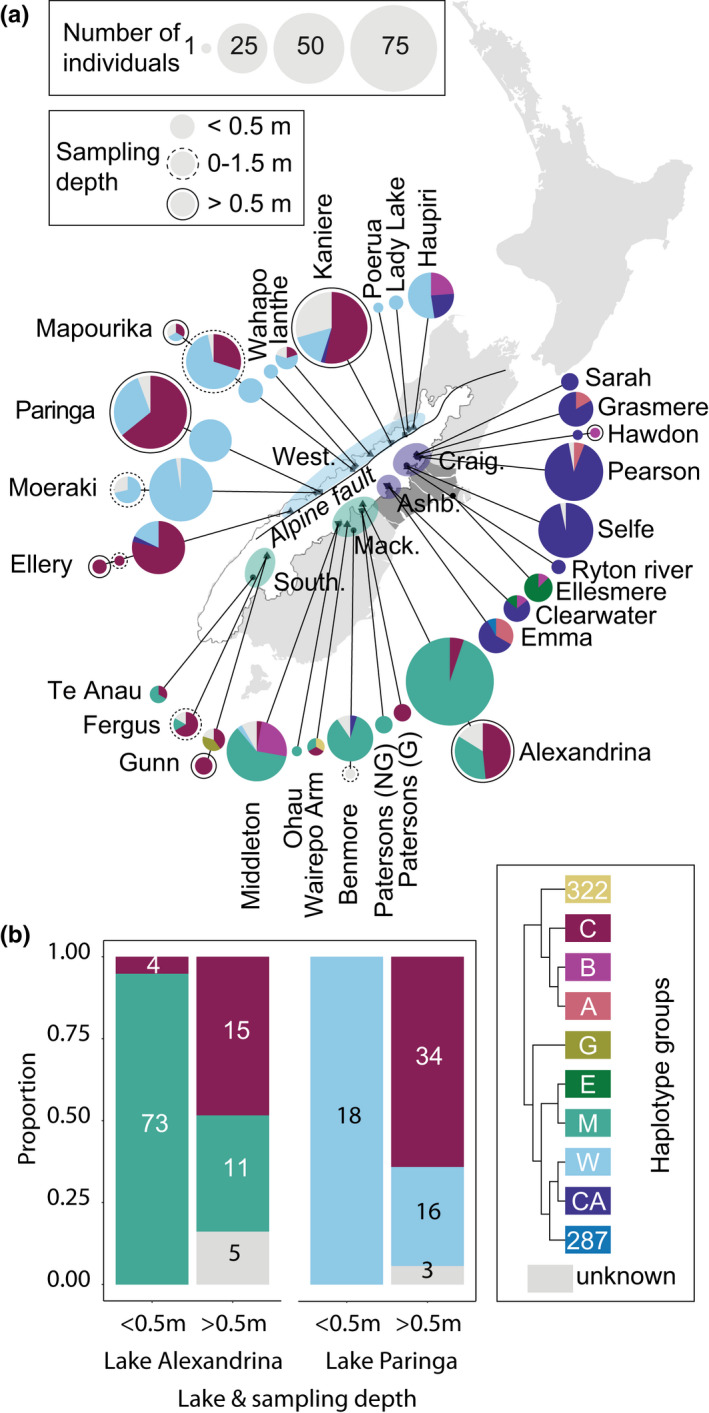
The geographical and depth distribution of *Atriophallophorus* spp. mitochondrial haplotype groups in lakes on New Zealand's South Island. (a) The geographic distribution of mitochondrial haplotype groups. Note the legend and the mitochondrial phylogeny in Figure 2a for phylogenetic relationships between the groups. Multiple samples from the same lake are categorized in three depth classes and the size of the pie charts represents sample size. During the last glacial maximum, an ice sheet covered the southern Alps: the white area, after James et al. (2019). At the same time an extensive area of gravel outwash was found in the central part of the island: the dark grey area, after Fleming (1975). These Pleistocene landscape features are hypothesized to have led to a distributional break in many New Zealand taxa, and is referred to as the *beech gap* due to the absence of continuous *Nothofagus* forest. The line represents the Alpine Fault, after Batt et al. (2000). Regions as mentioned in the text are: Mackenzie District (Mack.), Southland (South.), Ashburton Lakes (Ashb.), Craigieburn Range (Craig.) and West Coast (West.). Region colour reflects the dominant haplotype group. (b) The relative frequency of *Atriophallophorus* haplotype group C is higher in deeper habitat, indicating that this may represent an ecologically divergent *Atriophallophorus* species (see Figure 2 for species delimitation). Numbers within the bars indicate the sample size

Mountain uplift along the Alpine Fault (Figure 1a) initiated between c. 4 and 5 Mya and has since proceeded at a rate of c. 0.4 km/Myr (Batt et al., 2000; Jiao et al., 2017; Tippett & Kamp, 1995a,b). Throughout the latter half of these last 5 Ma, Pleistocene glacial cycles led to repeated periods of extensive glaciation. During the most recent glacial maximum, c. 30–18 Kya, glaciers extended along most of the Southern Alps (James et al., 2019; Shulmeister et al., 2019). The main biological refugia are thought to have formed in the northern and south‐eastern areas of the island, which remained free of ice (Fleming, 1975; Wallis & Trewick, 2009; Wardle, 1963). The refugia were separated by both the glaciated Southern Alps and a large area of gravel outwash in the Canterbury region to the east of the Southern Alps (see Figure 1a and Fleming, 1975). The contemporary southern West Coast was mostly glaciated (Fleming, 1975), but smaller biotic refugia may have existed in this area (Weir et al., 2016).

Many of the present‐day lakes around the Southern Alps were in contact with glaciers, or within the extent of the glaciers during the last glacial maximum (James et al., 2019; Sutherland et al., 2019). The post‐glacial colonization of these lakes by their contemporary communities may therefore depend on their proximity to past biotic refugia. One aquatic organism that is commonly found in these lakes, is the freshwater snail *Potamopyrgus antipodarum* (Grey, 1843). Pleistocene refugia have been implicated in shaping mitochondrial lineage distributions of *P. antipodarum*, as populations from the Southland region are genetically differentiated from more northern populations (Neiman & Lively, 2004; Paczesniak et al., 2013).

*Potamopyrgus antipodarum* hosts a diverse community of trematode parasites (Hechinger, 2012; Winterbourn, 1974), including *Atriophallophorus winterbourni* Blasco‐Costa et al. (2019) (formerly referred to as *Microphallus* sp.). This parasite has a two‐host life cycle, in which *P. antipodarum* is the intermediate host. The parasite reproduces clonally in the snail and fills its gonad with hundreds of cysts (Feijen, 2020). Trophic transmission to waterfowl such as dabbling ducks (*Anas platyrhynchos*, *Anas superciliosa* and their hybrids) or diving ducks (New Zealand Scaup; *Aythya novaeseelandiae*) is required to complete the parasite's life cycle (Lively & McKenzie, 1991; Osnas & Lively, 2011). The adult hermaphrodite worm reproduces sexually in the bird's gut (Blasco‐Costa et al., 2019). Snails infected by *A. winterbourni* were found to move to the shallow water margin, suggesting that the parasite induces snail migration to facilitate trophic transmission to dabbling ducks (Feijen, 2020). The interaction between *A. winterbourni* and *P. antipodarum* has long been of interest for the role of local adaptation, including frequency‐dependent adaptation by the parasite to locally common, clonal genotypes of *P. antipodarum* (Jokela et al., 2009; King et al., 2009; Lively, 1989; Lively et al., 2004).

Opposing processes may affect gene flow and local adaptation for a parasite (Gandon et al., 1996; Garant et al., 2007; Thompson, 1999). On the one hand, theory predicts that parasite migration promotes local adaptation, because gene flow may supply parasite populations with genetic variants that are advantageous in responding to novel resistance variants in local host populations (Garant et al., 2007; Slatkin, 1987). On the other hand, if gene flow between parasite populations is too high it may have a homogenizing effect and the expectation that a parasite population adapts on local resistance variants of its host population is no longer justified (Louhi et al., 2010; Slatkin, 1987). For parasites with multiple host life cycles, definitive host mobility has been implicated as determinant of parasite gene flow (Blasco‐Costa & Poulin, 2013) and has been suggested to negatively affect local adaptation to the intermediate host (Johnson et al., 2021). In contrast, *A. winterbourni* is an example of a parasite with strong local adaptation to its aquatic snail host, while having a flying definitive host and the associated high level of gene flow (Dybdahl & Lively, 1996; Lively, 1989). Dybdahl and Lively (1996) show that the pairwise genetic differentiation between host and parasite populations is positively correlated, but that the parasite lacks strong geographic structure (Wright's *F*_ST_ among lakes: 0.174 for the host and 0.017 for the parasite; based on allozyme loci).

It has become a favoured practice to combine different molecular markers to infer phylogeographic history. In theory, population subdivision should be stronger in mitochondrial genomes than in nuclear genomes, as maternal inheritance of the haploid genome reduces the effective population size four‐fold, thus making these markers more susceptible to drift (Birky et al., 1983; Birky Jr. et al., 1989). A disadvantage is that phylogeographic history may be oversimplified due to the faster fixation of mitochondrial variants (Zhang & Hewitt, 2003). Further complicating factors include discordant patterns or unexpectedly large differences between molecular markers in their power to infer geographic structure, due to processes like selection, introgression, incomplete lineage sorting or male‐biased dispersal (Bensch et al., 2006; Blower et al., 2012; Monsen & Blouin, 2003; Morales et al., 2015; Paczesniak et al., 2013; Pavlova et al., 2013; Toews & Brelsford, 2012; Wielstra & Arntzen, 2020). Combining both nuclear and mitochondrial data and an assessment of their congruence is thus the preferred method to make strong inferences about population history (Godinho et al., 2008; Zhang & Hewitt, 2003).

We revisited the population genetic structure of *A. winterbourni*, this time combining mitochondrial haplotype data with nuclear multilocus SNP genotypes. We found that *A. winterbourni* is part of a cryptic species complex and that *A. winterbourni* populations from different geographic regions exhibit strong mitochondrial divergence. By combining extensive sampling of the geographic range with molecular and ecological data, our data set allows for species delimitation and phylogeographic inference. We conclude that at least three and potentially more cryptic species need to be considered and that Pleistocene glaciation has most probably led to the phylogeographic patterns found for the most common of these species, *A. winterbourni*.

## 2 MATERIALS AND METHODS

### 2.1 Sample collection

*Potamopyrgus antipodarum* snails were collected from 33 lakes and two rivers (the majority during 2018). Snails were usually collected from shallow habitat while wading (<0.5 m depth). For several lakes, additional samples were collected from deeper macrophyte beds while free‐diving (up to 6 m). Samples were thus categorized in either shallow (0–0.5 m depth) or deeper (over 0.5 m depth) categories. In a few cases, snails were collected between 0 and 1.5 m depth and are thus placed in an intermediate depth category. Usually 100 random snails from each sample were dissected under a microscope (for a total of ca. 6000 snails), but in some populations we increased the sampling effort to collect more parasite individuals. Infected snails were stored in 100% ethanol until DNA extraction. A total of 574 infections were collected from 29 snail populations. From 16 of these populations, <10 individuals were collected and for six populations the infection frequency was too low to find any *Atriophallophorus* parasites. More details on sample sizes and locations are provided in Table S1.

### 2.2 Molecular data acquisition

For species delimitation, population genetic analysis and phylogeographic inference we collected two data sets: a partial sequence of the mitochondrial gene NADH5 and nuclear multilocus SNP genotypes. Additionally, a small subset of 35 individuals was chosen to sequence the taxonomic marker genes 28S and ITS2, to assess the relatedness of parasites from divergent mitochondrial lineages with these nuclear genes. For divergence dating purposes, we collected 28S and COI sequences from 15 Plagiorchiida species from NCBI Genbank, including two *Atriophallophorus* specimens from New Zealand (Table S2 and Figure S2) and sequenced COI for one individual. Details about the included individuals in each data set can be found in the Supporting Information data set and protocols are outlined below.

#### 2.2.1 DNA extractions

DNA was extracted from a single cyst per infected snail, using a custom designed extraction kit from LGC Genomics GmbH. Each cyst was isolated from preserved snail tissue, air‐dried, washed with molecular grade water (Sigma‐Aldrich) and pipetted into a solution containing 20 μl LGC PN lysis buffer and 2 μl LGC protease. The sample was lysed on a thermoshaker at 60°C for 2 h. Following lysis, 30 μl of LGC SB binding buffer and 2 μl of LGC magnetic bead suspension were added and the solution was incubated at room temperature for 20 min. The beads were subsequently washed with 50 μl of each of the LGC buffers BN1, TN1 and TN2. A final elution step was done by heating the beads at 60°C for 10 min with 20 μl LGC elution buffer AMP.

#### 2.2.2 Mitochondrial NADH5 sequencing

A 742 base pair fragment of NADH5 was amplified using polymerase chain reaction (PCR) with the Promega GoTaq G2 DNA polymerase kit. Initially the primers F2micND5: 5′‐*cttcaaccttggttgctgcc*‐3′ and R2micND5: 5′‐*tcccaacgaaacctaaaactgc*‐3′ were used. After a 2 min initial denaturing step (94°C), 35 PCR cycles (denaturing at 94°C for 30 s; annealing at 52.3°C for 1 min; extension at 72°C for 1 min) were followed by a 5 min final extension step (72°C). For some samples this protocol was not successful and the PCR was thus repeated with alternative primers MicND5longF: 5′‐*tygttggaagctatgcgtgc*‐3′ and MicND5longR: 5′‐*tgcgccrgttggctttac*‐3′. The PCR protocol was similar, except for annealing at 50.0°C. Amplicons were sequenced using the 3130xl DNA Analyser (Applied Biosystems) at the Genetic Diversity Centre at ETH Zürich (GDC). Forward and reverse sequences were assembled in Geneious Prime 2020.0.4 and manually checked for errors. NADH5 was successfully sequenced for 496 individuals (86.4%). For an additional 39 samples (6.8%), sufficient sets of diagnostic bases in low quality sequences allowed for haplotype group assignment.

#### 2.2.3 Multilocus SNP genotyping

Parasites were genotyped using 35 diallelic, synonymous SNP positions in coding genes. Details about the assay design, SNP positions, primers can be found in Table S3 and the results of a power analysis are provided in Figure S2. A set of 535 individuals was selected for genotyping with Fluidigm 96.96 dynamic array chips. The Fluidigm User Guide (PN 68000098 M2) protocol was followed, with three modifications to accommodate low DNA concentrations (<10 ng/μl): (I) 1.69 μl of the DNA extract was added to 5.6 μl Specific target amplification (STA) Pre‐Mix, (II) 22 STA PCR cycles and (III) STA product was diluted 50 times. Genotyping was successful for 520 individuals (97.2%).

#### 2.2.4 COI, 28S and ITS2 sequencing

Protocols are described in Blasco‐Costa et al. (2019) and amplicons were sequenced as described above. We successfully sequenced COI for one individual, ITS2 for 29 individuals and 28S for 28 individuals. Failure to get high quality 28S and ITS2 data was attributed to low primer specificity and, since the outer layer of a parasite cyst consists of snail tissue, the amplification of host DNA.

### 2.3 Phylogenetic analysis and species delimitation

**FIGURE 2:**
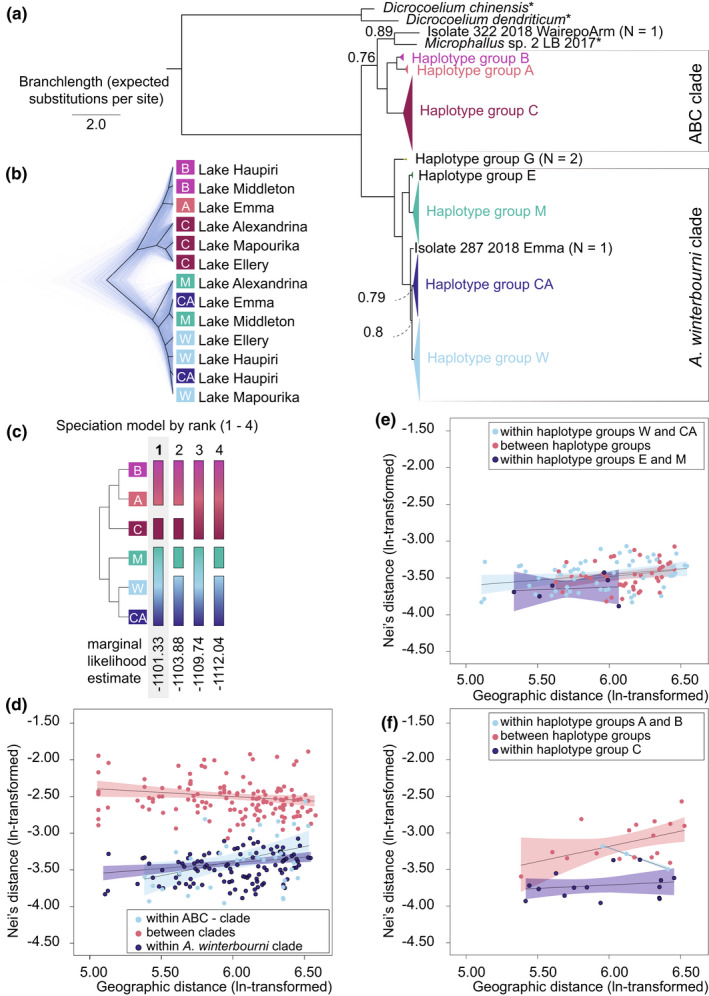
Evidence for speciation among *Atriophallophorus* specimens from the South Island of New Zealand. (a) Bayesian phylogenetic inference of unique mitochondrial NADH5 sequences. The 50% majority rule consensus tree is shown and branches of closely related sequences are collapsed. Node labels denote posterior probability where this is lower than 0.99. (b) Coalescent‐based species tree inference using multilocus SNP data from lakes where individuals from both major mitochondrial clades where found. Genetic structure reflects the mitochondrial phylogeny in (a), rather than the geographic sampling location. Mitochondrial haplotype groups are denoted with colours that match the mitochondrial phylogeny in (a). (c) Path sampling analysis with Bayes factor delimitation (BFD). Models are ordered from the left to right, with the highest support for the left‐most model (1). A three species model in which haplotype groups A and B belong to another species than haplotype group C is favoured over a two species model, while splitting of the *A. winterbourni* clade leads to a worse model fit. Mitochondrial haplotype groups are denoted with colours that match the mitochondrial phylogeny in (a). (d) Between‐clade pairwise genetic distance exceeds within‐clade pairwise genetic distance across the entire geographic range. Regression lines and 95% confidence intervals are estimated by analysing 1000 bootstrapped random samples with replacement to overcome the lack of independence of residuals for those pairs that share a population. (e) No support for further splitting of the *A. winterbourni* clade, all groups have same relationship between genetic and geographic distance. (f) Although sample size is small (only three population samples for haplotype groups A and B had at least four individuals), the ABC clade may consist of two separate species, since between group differentiation exceeds within group differentiation

The presence of deep divergence in the NADH5 phylogeny called for species delimitation analysis. Since a comparison of methods is important for accurate species delimitation (Carstens et al., 2013), we conducted two independent species delimitation analyses of multilocus SNP data to assess how divergence in the nuclear genome corresponds to the mitochondrial haplotype affinity. Clade and haplotype group definitions as discussed below are provided in the NADH5 phylogeny in Figure 2a. For species delimitation, we maintained a minimum sample size of four SNP genotypes belonging to individuals from a particular haplotype group from a lake, thus excluding isolates 287, 322, haplotype group G and any samples of haplotype groups from lakes that contained <4 individuals.

#### 2.3.1 Phylogenetic analysis

Our NADH5 data was complemented with sequence data from three additional specimens. *Dicrocoelium chinensis* (Sudarikov & Rizhdcov, 1951) and *Dicrocoelium dendriticum* (Rudolphi, 1819) were used as outgroup taxa (Genbank KF318786 and KF318787, respectively), while *Microphallus* sp. 2 LB‐2017 (Genbank SRR5170514) is a closely related *Atriophallophorus* specimen that was collected from *Potamopyrgus estuarinus* in New Zealand (Bankers & Neiman, 2017). Sequences were aligned with MAFFT version 7.450 (Katoh et al., 2002; Katoh & Standley, 2013) in Geneious prime® 2020.0.4 (https://www.geneious.com), using the translation align option (FFT‐NS‐i x1000 algorithm; BLOSUM62 scoring matrix; gap opening penalty of 1.53; offset value of 0.2). To facilitate alignment with the longer and divergent outgroup sequences, the full length NADH5 sequence from *A. winterbourni* from Zajac et al. (2021) was included during alignment. Unique sequences (*N* = 229) were extracted from the alignment for phylogenetic analysis. The GTR + I + G substitution model was selected using Akaike's information criterion (AIC) in jModeltest 2.1.10 (Darriba et al., 2012) and phylogenetic inference followed with MrBayes 3.2.7a (Ronquist et al., 2012) on the CIPRES Science Gateway (Miller et al., 2010). Default settings were used, except for four runs with four chains, a temperature parameter of 0.02 (to improve chain swap acceptance rates), a sampling frequency of 100 and diagnosis frequency of 500. The analysis ran for 6.258 million generations and was halted after the topological convergence diagnostic dropped below 0.01. Run diagnostics were assessed and 50% majority rule consensus tree were constructed after a burnin of 10%.

#### 2.3.2 Species delimitation

In a first analysis, we inferred a species tree from multilocus SNP data using a method based on the multispecies coalescent model. We supplemented this analysis with Bayes factor delimitation (BFD) for four alternative splitting scenarios. Both methods are implemented in the SNAPP 1.5.2 package in BEAST 2.6.5 (Bryant et al., 2012; Leaché et al., 2014). Since the coalescent method is computationally demanding, we selected a subset of 52 SNP genotypes from six lakes. Selection criteria for these lakes were that multiple mitochondrial lineages occur in sympatry and that they covered a wide geographic area. We thus selected the lakes Alexandrina, Benmore and Emma to the east of the Southern Alps and the lakes Haupiri, Mapourika and Ellery to the west of the Southern Alps (Figure 1a). From each lake we selected a set of four random individuals from each of two haplotype groups, with the exception of Lake Haupiri, where we sampled four individuals from each of three haplotype groups. Each set of individuals was assigned a unique population identifier and species tree inference was performed without prior information on the mitochondrial or geographic affinity of these sets of individuals. Default settings were applied, except for the mutation rates which were estimated before the analysis (*u* = 0.57, *v* = 3.78). We used a chain length of 300.000, a burnin of 10% and a sampling frequency of 10. Run diagnostics were assessed with Tracer version 1.7.1 (Rambaut et al., 2018). After construction of the species tree, we performed BFD by running a path sampling analysis on four different models of speciation: a three species model in which the ABC clade consists of two species (Figure 2c; model 1), a four‐species model in which both the ABC clade and the *A. winterbourni* clade are split (Figure 2c; model 2), a two species model in which the ABC clade and the *A. winterbourni* clade both represent a single species only (Figure 2c; model 3) and, finally, a three species model in which the *A. winterbourni* clade consists of two species (Figure 2c; model 4). The same data set and input file as in the species tree estimation was used, with the exception of the regrouping of individuals according to the four models and a log normal prior for λ (*M* = 1.2 and *S* = 1.2). Each analysis included eight steps, an initial chain length of 5000, a burnin of 10% and a preburnin of 100 generations. Each step was restarted until the effective sample size of the marginal likelihood was over 200 for each step of each species delimitation model.

In a second species delimitation analysis based on SNP data, we tested if within‐clade pairwise genetic distance between population pairs of either the *A. winterbourni* or the ABC clade was significantly lower than between‐clade population genetic distance. As pairwise genetic distance is expected to increase with geographic distance (IBD model), this can bias species delimitation based on genetic distance (Hausdorf & Hennig, 2020). We therefore analysed the differences in genetic distance while taking the geographic distance between population pairs into account. This analysis for species delimitation was adopted from Hausdorf and Hennig (2020) and conducted in IBM SPSS version 27 (SPSS, 2019). We constructed a pairwise genetic distance matrix between all sampling units (populations) as defined by lake and mitochondrial clade affinity (Figure 1a). Two sympatric haplotype groups from the same lake are thus assigned zero geographic distance. A minimum sample size of four specimens was maintained and loci with a minor allele frequency (MAF) over 0.01 were used (*N* = 31). We calculated Nei's genetic distance using the dist.genpop function in adegenet 2.1.5 (Jombart, 2008) and used a geographic distance that excludes migration routes above 1200 m in altitude (see the section on isolation by distance below for more information). This resulted in two distance matrices which we analysed using linear regressions combined with bootstrapping as described in Hausdorf and Hennig (2020). Bootstrapping is necessary to overcome the pseudo‐replication problem in regression analyses on pairwise distance matrices. We followed Hausdorf and Hennig (2020) by ln‐transforming both genetic and geographic distances to linearize the relationship between the two distances. Since some distances were zero, we added a 25% percentile to each distance before ln‐transformation (0.01570 and 157.7988 km for Nei's genetic distance and geographic distance, respectively), as recommended by Hausdorf and Hennig (2020).

Analysis of covariance was applied using the type of pairwise comparison as fixed grouping factor: genetic distances within the *A. winterbourni* clade, within the ABC clade or between clades. Geographic distance was treated as a continuous covariate to explain variance in genetic distance. The model was additionally fitted with an interaction term between the fixed grouping factor and the covariate, which fits one regression equation per fixed factor. The interaction term between geographic distance and group identity tests for a significant difference among the slopes of the three groups. When the slopes do not differ, the fixed effect of group factor tests for difference in the intercepts of each three regressions. When the slopes do differ, the model provides predictions with confidence intervals of differences between the groups at any given covariate value. We analysed the confidence intervals for statistical inference from 1000 bootstrapped samples (with replacement) of the data.

After analysis of the *A. winterbourni* clade versus the ABC clade, a similar analysis was performed within each of these two clades to test for evidence supporting further species splitting. Analyses were conducted separately on the two data sets. Individuals from both the *A. winterbourni* and ABC clades were grouped according to the most basal phylogenetic split within each clade. As some of the clades in these analyses had small sample size, we resorted to stratified bootstrapping to keep the sample size for each categorical group intact, using the “strata” option in IBM SPPS version 27 (SPSS, 2019).

### 2.4 Phylogeographic inference

Only *A. winterbourni* (the *A. winterbourni* clade; Figure 2a) was selected for analysis, since geographic coverage and sample sizes for other putative species were not sufficient. Three steps were used for phylogeographic inference. We tested if mitochondrial and nuclear population differentiation are concordant, then tested if isolation by distance was significant for both data sets and finally conducted phylogeographic analysis based on mitochondrial NADH5 data.

#### 2.4.1 Mitonuclear congruence

Congruence between nuclear and mitochondrial pairwise *F*_ST_ matrices was assessed using the CADM test and Kendall's coefficient of concordance (W) from the R package ape 5.1–1 (Kendall & Smith, 1939; Legendre & Lapointe, 2004; Paradis & Schliep, 2019). Pairwise *F*_ST_ matrices were calculated for both data sets using both Arlequin 3.5.2.2 (Excoffier & Lischer, 2010) and the Empirical Bayes method (EB*F*_ST_) from the R package FinePop 1.5.1 (Kitada et al., 2017). The latter method was included because it may provide higher precision of *F*_ST_ estimates under high gene flow scenarios with few loci (Kitada et al., 2017). Fifteen lakes with at least six *A. winterbourni* individuals were included (the complete data sets thus included 341 SNP genotypes and 343 for mitochondrial haplotypes). SNP loci with a MAF above 0.01 were included (*N* = 32). For NADH5, 109 unique sequences were found among 343 individuals due to rare substitutions. Highly similar sequences were therefore reassigned as follows: (i) pairwise distances (JC69) were calculated using phangorn 2.5.5 (Schliep, 2011); (ii) 22 sets of haplotypes were resolved using hierarchical clustering followed by group assignment using the hclust and cutree (height = 0.005) functions in R (R Core Team, 2020) and (iii) the first occurring sequence was selected to represent its group. Haplotype assignment is visualized in Figure S3. Default settings were used for both the Arlequin and Finepop *F*_ST_ etimates, with the exception of 1000 permutations and 100,000 iterations, respectively.

We then used Structure 2.3.4 (Hubisz et al., 2009) to infer population genetic structure in the SNP data set (32 loci with MAF > 0.01; minimum diploid sample size of six). Prior population information was used (lake identity) and correlated allele frequencies were assumed. We ran the analysis for all possible numbers of clusters from *K* = 1 to 15 (the number of lakes) for 100,000 generations after a burnin of 20,000, with three replicates for each value of *K*. Structure harvester version 0.6.94 was used to analyse the output and determine the optimal value of K (Earl & vonHoldt, 2011). Based on a comparison of mitochondrial haplotypes and the cluster affinity as determined with Structure 2.3.4, we repeated the CADM test for mitonuclear congruence, this time excluding three neighbouring populations with discordant nuclear and mitochondrial affinity. These populations could form a putative contact zone between divergent populations since they are situated in the former area of gravel outwash in the central part of the South Island (Lakes Clearwater, Emma and Selfe; Figure 1a).

#### 2.4.2 Isolation by distance

Tests for isolation by distance were also carried out using the CADM test with 10,000 permutations for significance testing (Legendre & Lapointe, 2004). The four nuclear and mitochondrial pairwise *F*_ST_ matrices (see above and Tables S4) were compared with a direct geographic distance matrix (geodesic distance) calculated with the geodist R package (Padgham & Sumner, 2020). In addition, a series of geographical distance matrices where used that incorporate topography and different maximum migration altitudes (Tables S5). These geographic distances were calculated using topographic path analysis with the topoDistance R package (Wang, 2020). Paths were modelled from entirely coastal routes up to nearly linear distances across the mountains. An altitude raster was employed as environmental cost raster, setting altitudes from 800 to 2200 m as maximum altitude, in 200 m increments to create eight different matrices. The remainder of the altitude raster was used to model environmental suitability, ranging from most suitable at sea level to least suitable at the maximum migration altitude in linear fashion. As such, topographic paths often follow valley bottoms and thus waterways. This is a preferred method for a parasite that alternates between aquatic snails and waterfowl as hosts.

#### 2.4.3 Marginal approximation of the structured coalescent

We conducted phylogeographic analysis using Mascot 2.1.2 in BEAST 2.6.5 (Bouckaert et al., 2019; Muller et al., 2018). Nine lakes with a sample size of 16 or higher were selected, and sample sizes were equalized through random sampling (*N* = 16). NADH5 sequences were aligned with MAFFT version 7.450 (Katoh et al., 2002; Katoh & Standley, 2013) in Geneious prime 2020.0.4 (FFT‐NS‐i x1000 algorithm; 100PAM/*K* = 2 Scoring matrix; gap opening penalty of 1.53; offset value of 0.2). The HKY + I model was selected with AIC in jModeltest 2.1.10 (Darriba et al., 2012) and ancestral node state probabilities and migration rates were inferred using Mascot 2.1.2. Default settings were used except for a uniform prior on the tree height. This prior matches the divergence dating estimate for the divergence of *A. winterbourni* lineages (0.04–1.16 Ma) 30 million; burnin 10%; Run diagnostics assessed in Tracer version 1.7.1 (Rambaut et al., 2018). Median migration rates, which are less sensitive to outliers than the mean in Markov Chain analyses, were evaluated in Tracer version 1.7.1 (Rambaut et al., 2018) and visualized using ggmap 3.0.0 (Kahle & Wickham, 2013).

### 2.5 Divergence dating

A data set of 28S and COI sequences from 15 Plagiorchiida was selected for divergence dating (Table S2). COI data was missing for five taxa. Three *Atriophallophorus* specimens from New Zealand were included: *A. winterbourni* (haplotype group M) from Blasco‐Costa et al. (2019), *Microphallus* sp. 2 LB‐2017 from Bankers and Neiman (2017) and novel data from *A. winterbourni* (haplotype group W), thus enabling the dating of the deepest split in the *A. winterbourni* clade. Sequences were aligned with MAFFT version 7.450 (Katoh et al., 2002; Katoh & Standley, 2013) in Geneious prime 2020.0.4. FFT‐NS‐i x1000 algorithm; 100PAM/*K* = 2 Scoring matrix gap open penalty of 1.53; offset value of 0.2. The HKY + G and GTR + I + G models were selected with AIC in jModeltest 2.1.10 (Darriba et al., 2012) for the COI and 28S data, respectively. Divergence dating followed using Beast2 version 2.6.3 (Bouckaert et al., 2019). A log‐normal calibration point was placed on the split between Gymnophallidae and *Proctoeces* (offset of 75.2 Ma [*M* = 1.0; *S* = 0.6]), since the oldest fossils attributed to Gymnophallids date back to the Late Campanian (76.2–75.2 Ma); see Huntley and De Baets (2015); Todd and Harper (2011) and Rogers et al. (2018) for more information. Default settings were used except for unlinked relaxed log‐normal clocks with exponential mean rates (mean = 10), a run length of 100 million generations, a 10% burnin and parameter logging and tree sampling every 10 thousand generations. Run diagnostics were assessed with Tracer version 1.7.1 (Rambaut et al., 2018) and a consensus tree was generated with TreeAnnotator version 2.6.3 (Bouckaert et al., 2019).

### 2.6 Ecological divergence of species

Fisher's exact test was used to test if the relative frequency of *Atriophallophorus* individuals from different mitochondrial lineages is affected by sampling depth. Only Lake Alexandrina and Lake Paringa were included, as those lakes had sufficient sample sizes (>18) from both shallow (<0.5 m) and deeper habitat (>0.5 m). Parasites with unknown mitochondrial affinity where excluded (<5% of the data; *N* = 8).

## 3 RESULTS

### 3.1 Phylogenetic analysis and species delimitation

After initial phylogenetic analysis of mitochondrial data, we used SNP data for species delimitation analyses to test whether monophyletic mitochondrial clades represent different species. We found evidence for the coexistence of at least three species among the common haplotype groups (Figure 2).

#### 3.1.1 Phylogenetic analysis

Within the NADH5 sequence data, pairwise sequence divergence was as high as 14.5%. This corresponds roughly to a maximum divergence of 10.2% COI that was found between the three New Zealand *Atriophallophorus* specimens in the divergence dating data set (Table S2). Apart from a few rare lineages, phylogenetic inference resolved the existence of two major mitochondrial clades, which could be subdivided into seven main haplotype groups (Figure 2a). Four of these seven were named after their disjunct geographical distributions as follows: group E (Lake Ellesmere), group M (lakes in, or bordering, Mackenzie District and Southland Lakes), group CA (high‐country lakes around the Craigieburn range and the Ashburton Lakes) and group W (West Coast Lakes). Together, these lineages form a monophyletic clade (posterior probability [PP]: 1.00). Since the *A. winterbourni* paragenophore (Blasco‐Costa et al., 2019) belongs to group M, we refer to this clade as the *A. winterbourni* clade. East of the Southern Alps, the border between the distributions of the most divergent *A. winterbourni* lineages coincides with the former area of gravel outwash in Canterbury District (Figure 1a). Three of the remaining main haplotype groups, A, B, and C, also form a monophyletic group in the phylogeny (PP: 1.00) and are jointly referred to as the ABC clade (Figure 2a). The closely related haplotype groups A or B where found in the Ashburton Lakes, the lakes around Craigieburn Range (Figure 1a), Lake Middleton and Lake Haupiri. In these lakes, the deeper habitats were not sampled, which may explain the seeming absence of haplotype group C (haplotype group C is associated with deeper habitat, see Figure 1b). Until more detailed surveys of deep habitats are obtained, we note that haplotype group C is shared between West Coast Lakes, Southland Lakes and Mackenzie district. In addition to these main haplotype groups, three rare lineages were found: (i) group G (*N* = 2, Lake Gunn; Figures 1a and 2a); (ii) one individual from Lake Emma (closely related to groups CA and W; Figure 2a) and (iii) one individual from Wairepo Arm that is related to *Microphallus* sp. 2 LB‐2017.

#### 3.1.2 Species delimitation

Two nuclear genes (ITS2 and 28S) were sequenced with the aim to aid species delimitation, but these genes were too conserved to resolve clades. No substitutions were found for ITS2 and one polymorphic base position was found in 28S, but this polymorphism was present in both major mitochondrial clades, defined in Figure 2a. The only substitution was found in 28S for *Isolate 322* (Figure 2a), but the sample size (*N* = 1) for this lineage is not sufficient.

Species delimitation with SNP data was more successful. The coalescent‐based species tree inference resolved a split between the ABC clade and the *A. winterbourni* clade that overrides geographical patterns (Figure 2b). The Bayes Factor Delimitation supported the three‐species model in which the ABC clade is split (Figure 2c, model 1) as the most likely model (marginal likelihood estimate [MLE]: −1101.33). The four‐species model was the second most likely model (Figure 2b, model 2; MLE: −1103.88), followed by the two species model (Figure 2c, model 3; MLE: −1109.74). The least likely model was the three species model in which the *A. winterbourni* clade was split (Figure 2c, model 4; MLE: −1112.04). Model 1 has positive support over model 2 (BF = 2 × [MLE] model 1‐MLE model 2) = 5.10) and decisive support (BF > 10 for both remaining MLE comparisons) over model 3 and model 4 (Kass & Raftery, 1995; Leaché et al., 2014).

Species delimitation using analysis of covariance indicated a similar result to the coalescent analysis. Population samples from the *A. winterbourni* and ABC mitochondrial clades were consistently divergent in the nuclear genome across the geographic range, even when found in the same lake (Figure 2d, Table S6.1). Pairwise genetic distances within each of the two major clades increased with geographic distance. The slopes of separate regressions were 0.166 (bootstrapped *p*‐value = .003; *R* ^2^ = 0.08) and 0.414 (bootstrapped *p*‐value = .012; *R* ^2^ = 0.15) for the *A. winterbourni* and ABC clade, respectively. The ANCOVA model indicated that these slopes did not differ significantly (*p* = .132, Table S6.1). In contrast, the between clade pairwise genetic distance decreased slightly with distance (slope = −0.101; bootstrapped *p*‐value = .048, *R* ^2^ = 0.03) and differed significantly from the within group slopes (*p* = .001, Table S6.1). For the analysis within the ABC clade, we found that distances within haplotype group C were smaller than distances within groups A and B or between group distances (Figure 2f; Table S6.2). Further splitting was not supported for the *A. winterbourni* clade, since between group genetic distances do not differ from within group genetic distances (Figure 2e; Table S6.3).

### 3.2 Phylogeographic inference

General concordance in geographic pattern was found between nuclear and mitochondrial data within the *A. winterbourni* clade and both data sets showed significant isolation by distance. Phylogeographic inference indicates that the two main genetically differentiated lineages of *A. winterbourni* arose in parapatry, the first south of the beech gap to the east of the Southern Alps, and the second either west of the southern Alps or north of the beech gap.

#### 3.2.1 Mitonuclear congruence

**FIGURE 3:**
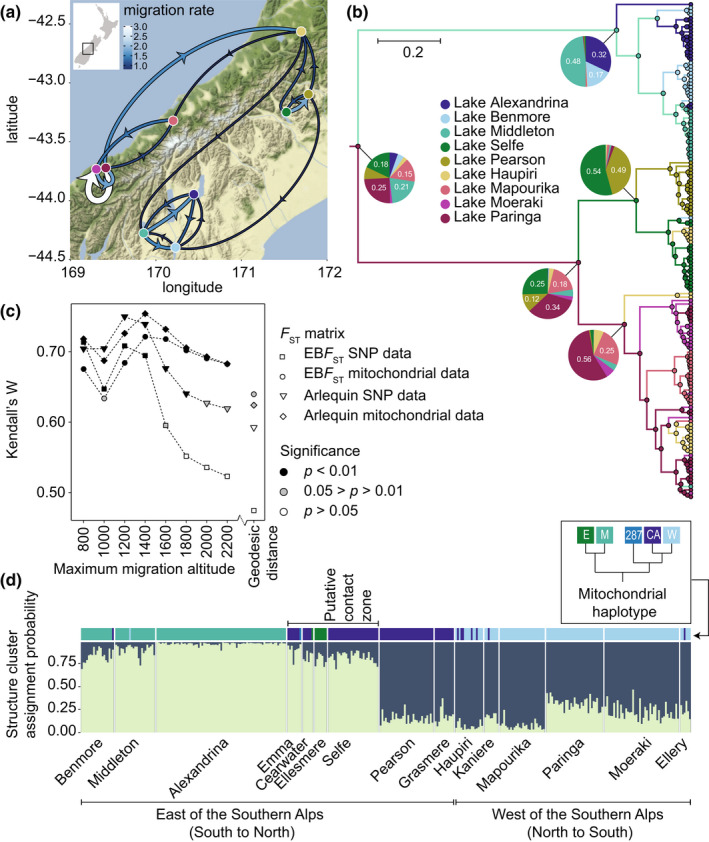
*Atriophallophorus winterbourni* phylogeographic inference and population structure. (a) The highest 25% of median migration rates between lakes are shown on a map of New Zealand (© OpenStreetMap contributors). The wider an arrow is, the more likely it is that a lineage within a particular lake originated from the direction from which the arrow originates. (b) State inference for ancestral nodes. Pie charts show state probability for the ancestral nodes, while node circles show the most likely state. Branch colours show the most likely state of the descendant node. (c) Tests for isolation by distance for both nuclear SNP data and mitochondrial data, using *F*_ST_ values calculated with both Arlequin and the empirical Bayes method. As geographic distance matrices both the direct (geodesic distance) and a series of distance matrices based on different maximum migration altitudes were employed (Figure S5). (d) Structure analysis resolved two clusters in the nuclear SNP data. In a putative contact zone between genetically divergent populations, discordance was found between mitochondrial and nuclear affinity. SNP data indicated that populations from the Lakes Emma, Clearwater and Selfe are more similar to neighbouring populations to the South, while their mitochondrial affinity is with neighbouring lakes to the North. Lakes are ordered counter‐clockwise around the Southern Alps, starting from the Southernmost lake to the east of the mountain range (see Figure 1a)

For *A. winterbourni*, population subdivision was considerably stronger (almost 20‐fold) for mitochondrial NADH5 data (global EB*F*_ST_: 0.39) than that of nuclear SNP data (global EB*F*_ST_: 0.02). Congruence between nuclear and mitochondrial based *F*_ST_ matrices was significant for EB*F*_ST_ (CADM test, Kendall's *W* = 0.69, *X* ^2^ = 143.73, *p* = .003), but not when using Arlequin (CADM test, Kendall's *W* = 0.59, *X* ^2^ = 122.36, *p* = .103). The structure analysis resolved two differentiated clusters of genotypes, one of which is associated with lakes west of the Southern alps and to the north of the beech gap, while the second consists of lakes south of the beech gap (Figure 3d). When excluding the three lakes in the beech gap region with differing mitochondrial and nuclear affinity (Lakes Selfe, Emma and Clearwater; Figure 3d), significant concordance emerged between mitochondrial and nuclear data for both the EB*F*_ST_ method (*W* = 0.78, *X* ^2^ = 101.87 and *p* = .001) and Arlequin (*W* = 0.65, *X* ^2^ = 85.09 and *p* = .033). Mitonuclear congruence of EB*F*_ST_ matrices is visualized with neighbour joining trees in Figure S4.

#### 3.2.2 Isolation by distance

When using a geodesic distance matrix, isolation by distance was significant for mitochondrial data (*p* < .05) but not for nuclear SNP data (*p* > .05). However, we found significant (CADM test: *p* < .05; Figure 3c) isolation by distance for both mitochondrial sequence data and nuclear SNP data when using distance matrices derived from topographic path modelling (Figure S5). For both data types, Kendall's W was highest (0.68 < *W* < 0.75; *p* < .003) for geographic distance matrices where maximum migration altitude was set to 1200–1400 m, after which Kendall's W decreases as increasingly direct cross‐alpine migration routes are allowed (Figure 3c).

#### 3.2.3 Marginal approximation of the structured coalescent

Using the Marginal approximation of the structured coalescent for state reconstruction and migration rate inference, we find that the ancestor of the *A. winterbourni* clade is not assigned to any geographic region with a high probability (Probabilities: 0.18 Lake Selfe, 0.25 Lake Paringa, 0.21 Lake Middleton and 0.15 Lake Mapourika; Figures 3a,b). However, the geographic origin of the two descendant lineages coincides with the beech gap hypothesis: the first originated east of the Alps, to the South of the beech gap (0.48 Lake Middleton, 0.17 Lake Benmore and 0.32 Lake Alexandrina) while the other originated either west of the Southern Alps, or east of the Southern Alps but to the North of the beech gap (0.34 Lake Paringa, 0.18 Lake Mapourika, 0.12 Lake Pearson, 0.25 Lake Selfe). Migration rates as inferred from the same analysis show that two barriers to mitochondrial gene flow exist for *A. winterbourni*: to the east of the Southern Alps, the migration rates are low between Ashburton District and Mackenzie District. To the South of the beech gap, where the Southern Alps are highest, the cross‐alpine migration rate is also low (Figures 3a,b).

### 3.3 Divergence dating

**FIGURE 4:**
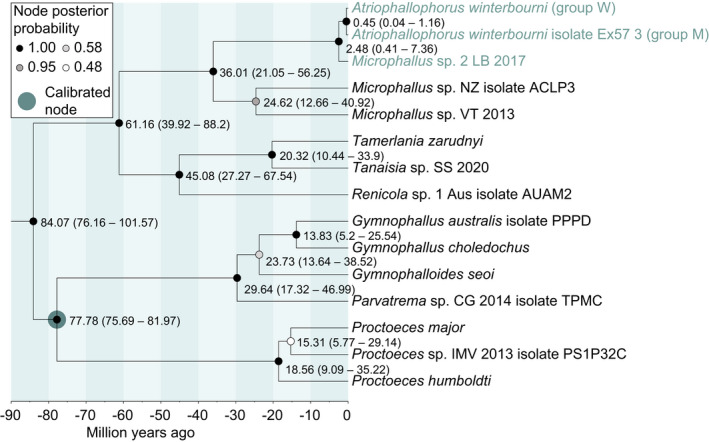
Divergence dating based on trematode COI and 28S sequence data. Node labels show the median divergence estimate and the 95% higher posterior density interval in brackets. The larger circle specifies the node that was constrained based on the fossil record. Based on current understanding of trematode taxonomy, *Microphallus* sp. 2 LB 2017 belongs to the genus *Atriophallophorus*. The split between the two *Atriophallophorus winterbourni* specimens represents the oldest split between *A. winterbourni* specimens, while the split between *Microphallus* sp. 2 LB 2017 and the two *A. winterbourni* specimens represents the oldest node among *Atriophallophorus* specimens in New Zealand (see Figure 2a for comparison with the NADH5 phylogeny)

The median estimated age of divergence between the most divergent *Atriophallophorus* lineages in the data set was 2.48 Ma (95% higher posterior density [HPD]: 0.41–7.36 Ma), while median estimated age of divergence within the *A. winterbourni* clade was estimated to have occurred 0.45 Ma (95% HPD: 0.04–1.16 Ma) (Figure 4).

### 3.4 Ecological divergence

In those lakes where haplotype group C and *A. winterbourni* coexist, haplotype group C increases in relative frequency towards the deeper habitat (Figure 1b). This was clearest for the larger samples from Lake Alexandrina and Lake Paringa (Figure 1c; Fisher's exact test for both lakes: *p* < .001).

## 4 DISCUSSION

The hypothesis that a common vicariant event during the Pleistocene glacial cycles has shaped the phylogeographic patterns of many taxa in the South Island of New Zealand has received increasing support, from taxa as diverse as birds, insect and plants (Trewick & Wallis, 2001; Wallis et al., 2016; Wallis & Trewick, 2009). Our analysis of the phylogeographic patterns of the most common *Atriophallophorus* species in New Zealand, *Atriophallophorus winterbourni*, shows that the same general process may have generated differentiation among populations of these trematode parasites. The characteristic distributional break in the central part of New Zealand's South Island (Fleming, 1975; Wallis et al., 2016; Wallis & Trewick, 2009; Wardle, 1963), was also recovered for divergent mitochondrial lineages of *A. winterbourni*. The most recent common ancestor of the *A. winterbourni* mitochondrial clade probably lived between 0.04 and 1.16 Ma ago (Figure 4), before the last glacial maximum (c. 0.018–0.030 Ma). From this ancestor, the *A. winterbourni* clade splits into two lineages, of which the first most probably originated in the South‐East of the South Island, while the second could have originated either to the west of the mountain range or in the North‐East of the South Island (Figure 3). In addition, cross‐alpine migration rates are low and isolation by distance is stronger when cross‐alpine migration routes are excluded (Figure 3). The most likely scenario that gave rise to the phylogeographic patterns of *A. winterbourni* is therefore genetic differentiation of populations in glacial refugia, while post‐glacial colonization of lakes was constrained by mountain ranges.

For *A. winterbourni*, significant concordance in pairwise *F*_ST_ values derived from nuclear and mitochondrial data was found and both data sets showed significant isolation by distance. In addition, the population structure in the nuclear DNA largely follows that of the mitochondrial haplotypes (Figure 3c). These similarities in population structure would suggest a similar phylogeographic history underlies the distributional patterns of both nuclear and mitochondrial genomes. However, a more comprehensive analysis may be obtained by comparing different phylogeographic methods on both the nuclear and mitochondrial data sets, such as nested clade analysis and approximate Bayesian computation (Bloomquist et al., 2010).

Isolation in biotic refugia followed by post‐glacial range expansion may result in secondary contact zones, where interbreeding between divergent lineages occurs (Toews & Brelsford, 2012). For the trematode *A. winterbourni* we found indication of high gene‐flow in the nuclear SNP data, in accordance with Dybdahl and Lively (1996), but strong geographic structure in mitochondrial data. Although SNP data and mitochondrial data differ in their degree of population differentiation, the geographic distribution of two genetically differentiated clusters of *A. winterbourni* nuclear genotypes largely matches the distribution of mitochondrial lineages (Figure 3d). An exception is found for three high country lakes east of the Southern Alps: lakes Selfe, Emma and Clearwater. These lakes are in an area that coincides with the former area of gravel outwash (Figure 1a) and that spans approximately 60 km along the length of the Southern Alps. Here nuclear SNP data indicates an affinity with southern populations while mitochondrial affinity is with northern populations (Figure 3d). Geographic mitonuclear concordance only became strongly significant after these three lakes were excluded from the concordance analysis. This area may thus represent a secondary contact zone where nuclear and mitochondrial gene‐flow is asymmetric.

Mitonuclear discordance in secondary contact zones and differences in the strength of nuclear and mitochondrial population differentiation may be explained by factors such as sex‐biased dispersal, sex‐biased offspring production, adaptive introgression or drift (Toews & Brelsford, 2012). Since *A. winterbourni* is hermaphroditic, sex‐biased gene‐flow is not a straightforward explanation. Sequential hermaphroditism with a delayed male function could explain higher gene flow in the nuclear genome, but contrary to this idea, in vitro studies suggest a delayed female function (Blasco‐Costa et al., 2019). Drift alone may be expected to generate stronger mitochondrial population structure as effective population size of mitochondria is one‐fourth of the nuclear genes. However, what we observe here is a 20‐fold higher mitochondrial *F*_ST_ which is more than one may explain by differences in effective population size between mitochondrial and nuclear genomes (Larsson et al., 2008). It is therefore more likely that adaptive introgression or asymmetric mitonuclear incompatibility are causing the observed patterns.

Apart from *A. winterbourni*, at least one other putative species of *Atriophallophorus* occurs in New Zealand. These species diverged relatively recently, between 0.4 and 7.4 Ma. Although mitochondrial divergence is strong between the two major mitochondrial clades (14.5% and 10.2% divergence in NADH5 and COI, respectively), we found that either insufficient time has passed to reach monophyletic lineages in the nuclear genome or rare introgression may occur. Most nuclear polymorphisms are shared between mitochondrial clades and no diagnostic SNP's were found. In such cases, species delimitation is challenging and ideally involves a combination of methods and types of data (Carstens et al., 2013; Shaffer & Thomson, 2007). The evidence for the existence of a reproductive barrier among *Atriophallophorus* haplotype groups lies in the observation that the genetic differentiation between sets of individuals from different mitochondrial clades is maintained in sympatry, across the entire geographic range (Figure 2). Both coalescent and regression methods for species delimitation are concordant in this study system and give strong support for at least three species among the common haplotype lineages. For the rare haplotype groups (Figure 2a) it is more difficult to delimit species, since geographic ranges do not overlap and sample sizes are small (Hausdorf & Hennig, 2020). The ecological divergence between specimens from different haplotype groups is also an indicator of speciation (Rissler & Apodaca, 2007). Although these individuals are sharing the same host species, the relative frequency at which genetically differentiated individuals are found changes with depth. The ecological divergence here may be indicative of adaptation to different definitive hosts. *A. winterbourni* may be common in the shallow because it manipulates the snail to move to habitats where transmission to dabbling ducks is likely (Feijen, 2020). If this trait is not present in the other species, it may be more likely to be transmitted to diving ducks.

To understand the processes that generate and maintain genetic diversity in natural populations it is essential to integrate ecological and geographic data with different types of genetic data. Our results show that, as in many other taxa in New Zealand, genetic differentiation among populations of the locally adapted *A. winterbourni* parasite bears a signature of Pleistocene glaciation and high mountain ranges. Although specimens from divergent lineages show little differentiation in the nuclear genome, our species delimitation methods are concordant and indicate that more than one species of *Atriophallophorus* is found in New Zealand freshwater lakes and that these species are ecologically divergent. At this time, it is difficult to assess to what causes the strong difference between nuclear and mitochondrial population differentiation for populations of *A. winterbourni*. Population structure of parasites with multiple‐host life cycles will not only be affected by the availability and mobility of their hosts, but also by the coevolving, reciprocally antagonistic interactions that may lead to rapid local adaptation.

## AUTHOR CONTRIBUTIONS

F.F. and J.J. conducted the fieldwork and dissected the snails. F.F. conducted molecular laboratory work, most analyses and wrote the manuscript. J.J. conducted the regression analysis in SPSS. J.J., N.Z., C.V. and I.B.‐C. were involved in interpreting the dataset, suggested further methods for analyses and data collection and collaborated in the writing process.

## CONFLICT OF INTEREST

The authors declare no competing interests.

### OPEN RESEARCH BADGES

This article has earned an Open Data Badge for making publicly available the digitally‐shareable data necessary to reproduce the reported results. The data is available at https://doi.org/10.5061/dryad.2ngf1vhnw.

## Supporting information


Appendix S1

## Data Availability

Sequences have been deposited in NCBI GenBank (accession nos. ON646781 through ON647334). The genotype data, alignments and input files for the analyses can be found on Dryad (https://doi.org/10.5061/dryad.2ngf1vhnw) (Feijen et al., 2022).
