# Supplementary material for: Phylogeography and cryptic species structure of a locally adapted parasite in New Zealand
Source: Mol Ecol. 2022 Jul 4;31(15):4112–26. doi: 10.1111/mec.16570 (PMC9541338; doi:10.1111/mec.16570)
Supplement: Supplementary file 1 — Appendix S1 [file MEC-31-4112-s001.docx]

Phylogeography and Cryptic Species Structure of a

Locally Adapted Parasite in New Zealand

Frida Feijen^1,2^*, Natalia Zajac^1,2,3^, Christoph Vorburger^1,2^,

Isabel Blasco-Costa^4,5^ and Jukka Jokela^1,2^

Supporting Information

*^1^Eawag, Swiss Federal Institute of Aquatic Science and Technology, Dübendorf, Switzerland.*

*^2^ETH-Zurich, Department of Environmental Systems Sciences, Institute of Integrative Biology, Zürich, Switzerland.*

*^3^Functional Genomics Center Zürich, ETH Zürich/University of Zürich, Zürich, Switzerland*

*^4^Natural History Museum of Geneva, PO Box 6434, CH-1211 Geneva 6, Switzerland.*

*^5^Department of Arctic and Marine Biology, UiT The Arctic University of Norway, Langnes, PO Box 6050, 9037 Tromsø, Norway.*

*Corresponding author: [ffeijen@ethz.ch](mailto:ffeijen@ethz.ch)

**Contents**

[Study System 3](#_Toc104911598)

[Data Acquisition 3](#_Toc104911599)

[Sample Collection 3](#_Toc104911600)

[**Table S1.** Summary of sampling depth and lakes. 4](#_Toc104911601)

[Divergence Dating Data 5](#_Toc104911602)

[**Table S2.** Taxa used in divergence dating. 5](#_Toc104911603)

[**Fig. S1.** Neighbour joining trees showing congruence between COI and 28S. 6](#_Toc104911604)

[Development and quality control of molecular markers 7](#_Toc104911605)

[NADH5 Primer design 7](#_Toc104911606)

[SNP assay design and methods 7](#_Toc104911607)

[SNP genotyping 7](#_Toc104911608)

[Power analysis 7](#_Toc104911609)

[**Fig. S2.** Power analysis for the 32 SNP loci. 8](#_Toc104911610)

[**Table S3.** Assay summary for nuclear SNP loci. 9](#_Toc104911611)

[*F*_ST_ Analyes 10](#_Toc104911612)

[**Fig. S3.** Haplotype assignment for *F*_ST_ analysis. 10](#_Toc104911613)

[**Table S4.1.** Pairwise *F*_ST_ tables using Arlequin. 11](#_Toc104911614)

[**Table S4.2.** Pairwise *F*_ST_ tables using EB*F*_ST_. 12](#_Toc104911615)

[**Fig. S4.** Neighbour-Joining trees showing mito-nuclear congruence. 13](#_Toc104911616)

[Geographic distance matrices 14](#_Toc104911617)

[**Fig. S5.** Topographic path analysis. 14](#_Toc104911618)

[**Table S5.1.** Geographic distance matrix at a maximum migration altitude of 800 m 15](#_Toc104911619)

[**Table S5.2.** Geographic distance matrix at a maximum migration altitude of 1000 m 15](#_Toc104911620)

[**Table S5.3.** Geographic distance matrix at a maximum migration altitude of 1200 m 16](#_Toc104911621)

[**Table S5.4.** Geographic distance matrix at a maximum migration altitude of 1400 m 16](#_Toc104911622)

[**Table S5.5.** Geographic distance matrix at a maximum migration altitude of 1600 m 17](#_Toc104911623)

[**Table S5.6.** Geographic distance matrix at a maximum migration altitude of 1800 m 17](#_Toc104911624)

[**Table S5.7.** Geographic distance matrix at a maximum migration altitude of 2000 m 18](#_Toc104911625)

[**Table S5.8.** Geographic distance matrix at a maximum migration altitude of 2200 m 18](#_Toc104911626)

[Species delimitation analysis using analysis of covariance 19](#_Toc104911627)

[**Table S6.1.** Bootstrapped parameter estimates (1000 samples) of the analysis of covariance. 19](#_Toc104911628)

[**Table S6.2.** Bootstrapped parameter estimates for the ABC clade. 19](#_Toc104911629)

[**Table S6.3**. Bootstrapped parameter estimates for *A. winterbourni* clade. 19](#_Toc104911630)

# Study System

The parasite *A. winterbourni*, formerly referred to as *Microphallus* sp. (Blasco-Costa et al., 2019), has a two-host life cycle. The snail *P. antipodarum* serves as an intermediate host and waterfowl serve as definitive host (Lively & McKenzie, 1991). A parasite egg needs to be ingested by the snail, after which the parasite produces hundreds of genetically identical larval stages (Feijen, 2020). These encyst as metacercariae within the host’s gonad and castrate the snail. Infected snails need to be ingested by waterfowl. Important hosts are dabbling ducks (European Mallard, *Anas platyrhynchos* Linnaeus, 1758; Grey Duck, *Anas superciliosa* Gmelin, 1789; Mallard-Grey Duck hybrids), or diving ducks (New Zealand scaup, *Aythya novaeseelandiae* (Gmelin, 1789)), see Osnas and Lively (2011). Snails infected with *A. winterbourni* were found to move to the shallowest littoral habitat in an experimental setting, suggesting that the parasite induces snail migration to shallow water to facilitate trophic transmission to dabbling ducks (Feijen, 2020). The metacercariae hatch in the gut of the definitive host and develop into adult hermaphroditic worms (Lively & McKenzie, 1991). Despite the transmission of hundreds of genetically identical hermaphroditic worms to a single bird, populations of *A. winterbourni* show no evidence of inbreeding (Feijen, 2020).

# Data Acquisition

## Sample Collection

*Potamopyrgus antipodarum* snails were collected from 33 lakes and 2 rivers (the majority during 2018). Rivers and many lakes were visited only once and a single shallow water sample of snails was taken. For some lakes, we visited the lakes twice or we took samples from different habitats. We usually dissected at least 100 random snails per sample for each lake or river, to estimate prevalence of infection. For six samples we dissected less than 100 snails, either because we already had previous samples from the lake or because a large enough sample of snails was hard to obtain. For some lakes we dissected additional snails to increase parasite sample size, but stopped counting the uninfected snails to increase time efficiency. No *Atriophallophorus* infections were found in five lakes and one river. An overview of lakes, sampling years and depths can be found in Table S1 and more details can be found for each parasite individual in the Supplementary Data (SD1).

### **Table S1.** Summary of sampling depth and lakes.

For more specific details on habitat and depth per sample see Supplementary Data SD1.

| **Region in text** | **District** | **Population** | **longitude** | **latitude** | **N (<0.5 m)** | **N (>0.5 m)** | **N (0 – 1.5 m)** |
| --- | --- | --- | --- | --- | --- | --- | --- |
| Ashburton Lakes | Ashburton District | Lake Clearwater | 171.04488 | -43.607345 | 7 | - | - |
|  |  | **Lake Emily*** | 171.22718 | -43.553385 | 0 | - | - |
|  |  | Lake Emma | 171.09911 | -43.627893 | 12 | - | - |
| Central Otago | Central Otago | **Lindis River*** | 169.52832 | -44.613486 | - | - | 0 |
| Craigieburn Range | Selwyn District | **Lake Georgina*** | 171.56622 | -43.31629 | 0 | - | - |
|  |  | Lake Grasmere | 171.77455 | -43.062964 | 13 | - | - |
|  |  | Lake Hawdon | 171.84992 | -43.100887 | 1 | 1 | - |
|  |  | **Lake Lyndon*** | 171.70527 | -43.299118 | 0 | - | - |
|  |  | Lake Pearson | 171.78284 | -43.098983 | 34 | - | - |
|  |  | Lake Sarah | 171.77543 | -43.051368 | 3 | - | - |
|  |  | Lake Selfe | 171.52513 | -43.246638 | 30 | - | - |
|  |  | Ryton River | 171.54159 | -43.278177 | 2 | - | - |
| Ellesmere | Selwyn District | Lake Ellesmere | 172.66193 | -43.79203 | 8 | 0 | 0 |
| Hurunui District | Hurunui District | **Lake Tennyson*** | 172.73949 | -42.210649 | - | 0 | - |
| Mackenzie District | Mackenzie District | Lake Alexandrina | 170.45072 | -43.942021 | 77 | 31 | - |
|  |  | Patersons Ponds (Glacial) | 170.43118 | -44.056012 | 3 | - | - |
|  |  | Patersons Ponds (Non-Glacial) | 170.43033 | -44.057351 | 3 | - | - |
|  | Mackenzie / Waitaki District | Lake Benmore | 170.22729 | -44.400064 | 21 | - | 1 |
|  |  | Lake Ohau | 169.86316 | -44.287427 | 1 | - | - |
|  | Waitaki District | Lake Middleton | 169.85054 | -44.281011 | 36 | - | - |
|  |  | Wairepo Arm | 170.07102 | -44.300208 | 3 | - | - |
| Queenstown-Lakes District | Queenstown-Lakes District | **Lake Hayes*** | 168.81541 | -44.969967 | 0 | - | - |
| Southland District | Southland District | Lake Fergus | 168.10517 | -44.848835 | - | - | 6 |
|  |  | Lake Gunn | 168.10083 | -44.858708 | 5 | 3 | - |
|  |  | Lake Te Anau | 167.7598 | -45.219685 | 3 | - | - |
| West Coast | Grey District | Lady Lake | 171.58049 | -42.598941 | 2 | - | - |
|  |  | Lake Haupiri | 171.69756 | -42.564438 | 21 | - | - |
|  | Westland District | Lake Ellery | 168.67848 | -44.066719 | 28 | 2 | 1 |
|  |  | Lake Ianthe | 170.6376 | -43.049797 | 5 | - | - |
|  |  | Lake Kaniere | 171.14381 | -42.836789 | - | 51 | - |
|  |  | Lake Mapourika | 170.20132 | -43.316777 | 6 | 3 | 30 |
|  |  | Lake Moeraki | 169.30114 | -43.733052 | 40 | - | 7 |
|  |  | Lake Paringa | 169.41119 | -43.721387 | 18 | 53 | - |
|  |  | Lake Poerua | 171.49405 | -42.704953 | 1 | - | - |
|  |  | Lake Wahapo | 170.27043 | -43.254899 | 2 | - | - |

*Lakes were no *Atriophallophorus* spp. infections were found.

## Divergence Dating Data

A dataset with 15 trematodes (Plagiorchiida) was constructed for divergence dating of New Zealand *Atriophallophorus* lineages (Table S2). Within Gymnophallidae, no individuals were found on GenBank for which both 28S and the required partial sequence of COI were available. We therefore combined COI and 28S from different individuals of *Gymnophallus choledocus*, to avoid having no COI coverage for this entire family. COI and 28S sequences from two different *A. winterbourni* individuals from Lake Mapourika were also combined because (I) both individuals shared an identical NADH5 sequence, which is linked to COI on the mitochondrial genome and (II) 28S was not found to vary for *Atriophallophorus* specimens in this study, except for one shared polymorphism between all major mitochondrial lineages. We first confirmed the congruence of COI and 28S genes in our dataset with neighbour joining trees (Fig. S1). These were generated in Geneious prime® 2020.0.4 (<https://www.geneious.com>) using default settings. In order to root the phylogeny and place the fossil calibration point (see main text), we compared these trees with the current understanding of phylogenetic relationships among trematodes (Pérez-Ponce de León & Hernández-Mena, 2019).

### **Table S2.** Taxa used in divergence dating.

Genbank Accession Numbers are provided for both COI and 28S. **Microphallus* sp. 2 LB-2017 belongs to the genus *Atriophallophorus* according to the most recent taxonomic insights.

| **Species** | **28S** | **COI** |
| --- | --- | --- |
| *Atriophallophorus winterbourni* (Lake Mapourika) | New | New |
| *Atriophallophorus winterbourni* isolate Ex57_3 (Lake Alexandrina) | MN342153.1 | MN342156.1 |
| *Microphallus* sp. 2 LB-2017* | SRR5170514 | SRR5170514 |
| *Gymnophalloides* *seoi* | KM246858.1 | - |
| *Gymnophallus* *australis* isolate PPPD | KM246854.1 | - |
| *Gymnophallus* *choledochus* | KM268112.1 | MN547969.1 |
| *Microphallus* sp. NZ isolate ACLP3 | KJ868216.1 | KJ868203.1 |
| *Microphallus* sp. VT-2013 | KF738451.1 | KF738454.1 |
| *Parvatrema* sp. CG-2014 isolate TPMC | KM246856.1 | - |
| *Proctoeces* *humboldti* KY432604 | KY432604.1 | - |
| *Proctoeces* *major* KX671307 | KX671307.1 | - |
| *Proctoeces* sp. IMV-2013 isolate PS1P32C | KY432617.1 | KY432632.1 |
| *Renicola* sp. 1 Aus isolate AUAM2 | KP903411.1 | KP903421.1 |
| *Tamerlania* *zarudnyi* | MW131090.1 | MW334947.1 |
| *Tanaisia* sp. SS-2020 | MW139645.1 | MW334948.1 |


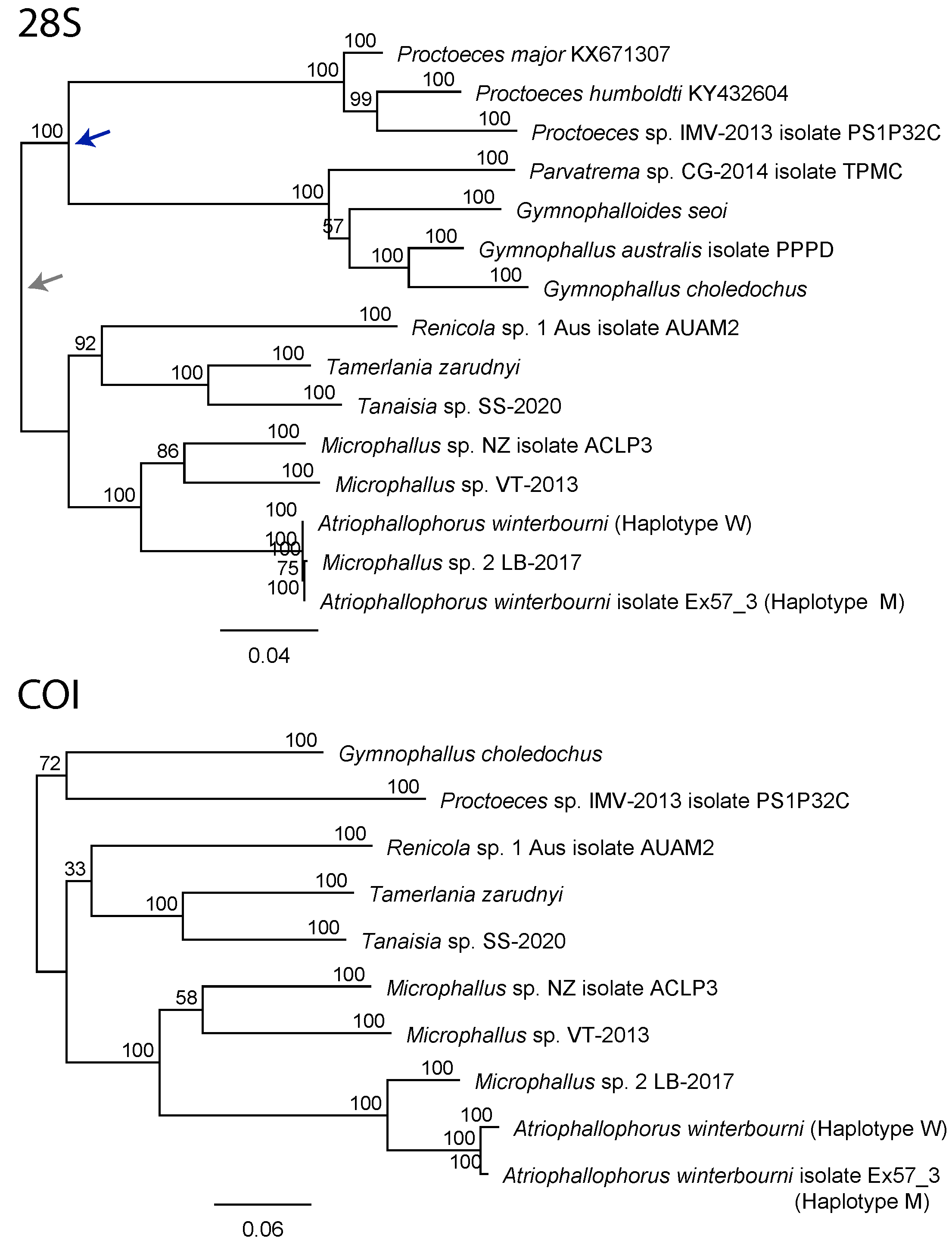


### **Fig. S1.** Neighbour joining trees showing congruence between COI and 28S.

Branch labels indicate bootstrap support. The grey arrow indicates where the phylogeny was rooted for divergence dating, while the blue arrow indicates the node that was calibrated using a lognormal distribution and offset of 75.2 Mya (M=1.0; S=0.6).

*Microphallus* sp. 2 LB-2017

A closely related New Zealand *Atriophallophorus* parasite was collected from *Potamopyrgus estuarinus* Winterbourn, 1970 by Bankers and Neiman (2017) for transcriptome analysis (NCBI Genbank sequence read archive SRR5170514). As *A. winterbourni* was previously referred to as *Microphallus* sp. (Blasco-Costa et al., 2019), this parasite is named *Microphallus* sp. 2 LB-2017. Due to recent taxonomic insights, *Microphallus* sp. was assigned to the genus *Atriophallophorus* and described as *A. winterbourni* (Blasco-Costa et al., 2019). Given the sequence data from *Microphallus* sp. 2 LB-2017 that is used in this study, this individual also belongs to *Atriophallophorus*, but the name as noted on Genbank is maintained to avoid confusion. We extracted sequence data from *Microphallus* sp. 2 LB-2017 for the genes NADH5, 28S and COI. Reads were obtained with a blast search of the Genbank sequence read archive SRR5170514, using our own data as reference sequence. Resulting reads were assembled de novo in Geneious prime 2020.0.4 and the consensus sequence was extracted.

# Development and quality control of molecular markers

## NADH5 Primer design

Primers F2micND5: 5’‑*cttcaaccttggttgctgcc*‑3’ and R2micND5: 5’‑*tcccaacgaaacctaaaactgc*‑3’ were designed based on the *A.* *winterbourni* transcriptome (NCBI Sequence Read Archive accession SRR5170515) from Bankers and Neiman (2017). For the design of alternative primers MicND5longF: 5’‑*tygttggaagctatgcgtgc*‑3’ and MicND5longR: 5’‑*tgcgccrgttggctttac*‑3’, data from *Microphallus* sp. 2 LB-2017 was added to find more conserved priming sites (NCBI Sequence Read Archive SRR5170514).

## SNP assay design and methods

SNP genotyping assays were based on *Atriophallophorus* transcriptomes (Genbank read archives SRR5170515 and SRR5170514) from Bankers and Neiman (2017). Reads were mapped to coding nuclear genes using standard settings in Geneious R10 to find diallelic, synonymous SNP positions. Genotyping assays (N = 48) were designed by the Fluidigm Corporation (South San Francisco, California, United States). Out of 48 SNP loci, 13 where rejected because automatic call rates and/or mean call confidence levels fell below 80%. The sequences, SNP positions and primers for the 35 successful assays can be found in Table S3.

## SNP genotyping

Parasites were genotyped with Fluidigm 96.96 dynamic array chips, at the GDC. Each SNP assay was replicated twice on each chip. Multilocus SNP genotyping was not attempted for 39 out of 575 DNA extractions because: (I) we ran out of extracted DNA (N = 8), (II) the sample size was either too low or already sufficient for population genetic analysis (N = 14) or (III) NADH5 sequencing had previously failed (N = 17). A manual check for wrongly assigned clusters or individuals was performed and genotypes with less than 90% locus assignment (N = 15) were removed with the Fluidigm SNP genotyping software, version 4.5.1. The SNP dataset thus included 520 individuals.

## Power analysis

We conducted a power analysis of our SNP dataset to infer whether or not the dataset is sufficient to infer significant population differentiation under high gene-flow scenarios. We conducted this analysis by simulating population divergence using POWSIM v.4.1 (Ryman & Palm, 2006). We used the 32 SNP loci that have a minor allele frequency of at least 0.01 within the *A. winterbourni* clade (Fig. 2A). For each simulation, 2 populations with an effective population size of 500 were allowed to drift apart for 10 to 50 generations (in increments of 10). Using 250 replications of each run. We repeated this analysis, but changed samples sizes of diploid genotypes per population (sample sizes of 5, 10, 15, 20, 30, 40 and 50 where used) for *F*_ST_ and significance assessment. To test for the alpha error, we repeated the same sampling series but at 0 generations of drift. We found that these loci should be sufficient to test for population divergence at the low *F*_ST_ values that we found between geographic regions in our study (0.02–0.05; Table S6.1). The results are shown below in Fig. S2.


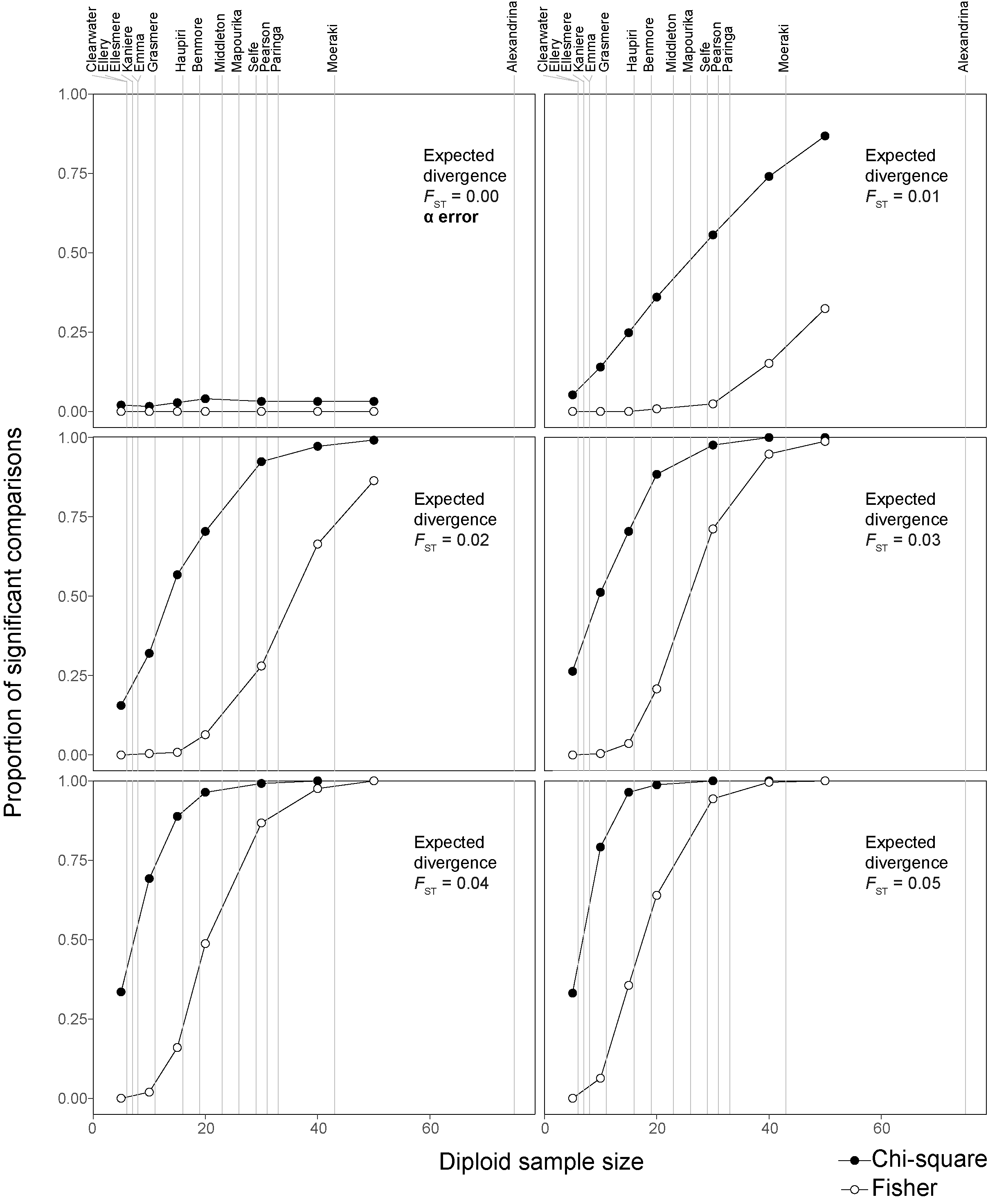


### **Fig. S2.** Power analysis for the 32 SNP loci.

Given the dataset with 32 diallelic SNP loci with a minimum allele frequency above 0.01, these figures sow the probability of α-error (top left: inference of significant divergence in non-diverged populations) and the power of both Chi-square and fisher test for different levels of population divergence (other panels; see expected *F*_ST_). The vertical lines show the sample sizes of the diploid genotypes from real populations in the study, as a reference.

### **Table S3.** Assay summary for nuclear SNP loci.

The locus name corresponds to the transcriptome assembly by Bankers and Neiman (2017). The assays were designed by the Fluidigm Corporation (South San Francisco, California, United States). Each locus is diallelic. The position in the sequence (pos.) and alternative alleles are given (a1 and a2). Before genotyping the target sequence was amplified in a PCR reaction with the Locus Specific Primer (LSP) and the Specific Target Primer (STA). The two Allele Specific Primers (ASP1 and ASP2) were used together with the LSP primer for genotyping.

| **Locus name** | **Pos.** | **a1** | **a2** | **ASP1 primer** | **ASP2 primer** | **LSP primer** | **STA primer** |
| --- | --- | --- | --- | --- | --- | --- | --- |
| GFFK01000628.1 | 330 | A | G | CGCAAAATTCAACGTAAAAAATTGCGA | GCAAAATTCAACGTAAAAAATTGCGG | GCAACCATCACCTTGAAACGATCA | ACTCCTTGGGCTCGCA |
| GFFK01000643.1 | 225 | A | T | CATCTTGTGCCGCCAACATT | ACATCTTGTGCCGCCAACATA | AGCCGATCGCGATGAAGCA | AGTGTTTTACACTTTTCTGCAACA |
| GFFK01000653.1 | 327 | C | T | CAAACTCTTCCTACGCGGC | GCAAACTCTTCCTACGCGGT | GAGGGTTTCGTACCACCAGGA | TGAAGTTCCTCGTCCCGG |
| GFFK01000716.1 | 138 | C | A | CCTCTGCCGTTCGTGTCC | CCTCTGCCGTTCGTGTCA | CTGGCTCATTCATGCAAGCCT | GCAATCGCCAAATTGGATCCTAA |
| GFFK01001005.1 | 538 | C | T | GCTTGGATCCTGAGGTAGCAG | GCTTGGATCCTGAGGTAGCAA | CGACAGCCAGATTTCACAATGCC | GCAAACAGACGACGGTTGC |
| GFFK01001006.1 | 777 | C | T | GCGATATTGCCACACCGGTAG | GCGATATTGCCACACCGGTAA | GATCACAGTTTGGTCTGCCCC | CTTTGATAGTCCAAAATGGCCG |
| GFFK01001194.1 | 60 | A | G | GGCGCTTCGCATTTCTTGT | GGCGCTTCGCATTTCTTGC | AATCAGGCAGCCATTAAAGAGC | GGTCAAGCATCTGAGCCAG |
| GFFK01001348.1 | 87 | C | T | GTATGCCACCTGTTGTTCCC | GGTATGCCACCTGTTGTTCCT | AACAACCATTGGAGCAGCACC | CACCGGGCTTCATCGTT |
| GFFK01001829.1 | 702 | T | C | CCCAGGGTCTTGTACAAGCT | CCCAGGGTCTTGTACAAGCC | CCATGTTGATCTGCACAGCGA | TTCTTCAATAAACAAATGCGTGCC |
| GFFK01001837.1 | 147 | C | T | CTTCGTCTTCAAAATCTACGTATGCG | TCTTCGTCTTCAAAATCTACGTATGCA | ACTGGCGTCCGAATGATCAGA | AGCCTGCAAAGATTCTTCGT |
| GFFK01002052.1 | 531 | A | G | GTTGAGCGAAAGCACCAGTAAA | GTTGAGCGAAAGCACCAGTAAG | AATGAGCTGCGCAATGCTCC | CGCGAGGCATACTTCCG |
| GFFK01002195.1 | 507 | C | T | ATACGATGTGTTGTTGCGCATC | ATACGATGTGTTGTTGCGCATT | GCCAAGCTGTCCGTTTACGT | CTTCACGATGGAAATGGATGCA |
| GFFK01002222.1 | 126 | A | T | AATCCCATACAGATCCTGGGTTT | CAATCCCATACAGATCCTGGGTTA | TCCGGCTGGGACCATTCT | GCAGCGAAAGCAGTTCGT |
| GFFK01002372.1 | 567 | C | T | AAGGACTTGATGAAATCATTCGAAACC | GAAGGACTTGATGAAATCATTCGAAACT | TTGCCATCACCTTTCAAGCA | CTGTGACTTTGTATCACCGAAGG |
| GFFK01002537.1 | 576 | C | T | AGTTCGAATGCTCCAAAGTTGTG | CAGTTCGAATGCTCCAAAGTTGTA | TGGAGCCATATAATTCCATCCTAACAACA | GGCTTCATTATCAACCATAAAAGCA |
| GFFK01002583.1 | 780 | C | T | TGCCTGGCACGTTACCG | TGCCTGGCACGTTACCA | ACGTCAAAAGGTCTGTGCAA | CAAGCGTATACGCTGACACT |
| GFFK01002700.1 | 183 | G | A | CTCGTACAACTGCATGTTCTGC | TCTCGTACAACTGCATGTTCTGT | TGTGGCGAACACGGTGACA | AAGTTCATAACCATTTCGAATCTCG |
| GFFK01002801.1 | 459 | A | C | CCACTGTTCTACCTGTCCCA | CCACTGTTCTACCTGTCCCC | ACGGATTATGAGCGTTTGCTGC | TTCTGGAGGGTCGACACC |
| GFFK01002993.1 | 261 | G | A | ATCCCTGATTTTGCTTGGCAC | CATCCCTGATTTTGCTTGGCAT | TGAGTGCATCCCCCAGGC | CGTCTTTCCCATCCCTGAT |
| GFFK01002994.1 | 420 | G | C | TTTTGGTCGCAACCAACCTG | CTTTTGGTCGCAACCAACCTC | CCCGTTCTATGTCCATTCCAC | CGTCTTTTGGTCGCAACC |
| GFFK01003107.1 | 252 | G | A | GACAAACTCAAAGCTGAACGTGAG | GACAAACTCAAAGCTGAACGTGAA | CCCAGGAGCATCGATGACAGTA | ACGCCTGGGTATTGGACA |
| GFFK01003309.1 | 1017 | C | T | TCCACTTCTGCTCCAACCAAC | TCCACTTCTGCTCCAACCAAT | ACCGACTGGAGAAACTCCACG | CGAAGCCAACCACAGCT |
| GFFK01004103.1 | 930 | C | T | GGGATTGACGTGAGCCCC | GGGATTGACGTGAGCCCT | CGATTGTTTCCACATGCGACTGTT | GGCTCATGACGCGGAAATT |
| GFFK01005746.1 | 219 | C | T | TGAGACATCCCATGATGTTCTCAAAG | TGAGACATCCCATGATGTTCTCAAAA | GACGGCTGAACGCTTTATTCGA | GGCGGTGAGACATCCC |
| GFFK01005813.1 | 51 | C | A | TTCGGTAAAATGCCTTTCATTTCCC | CTTCGGTAAAATGCCTTTCATTTCCA | GGTCACGTAGCGCACTCG | GGCTCTTCGGTAAAATGCCT |
| GFFK01006348.1 | 1599 | C | T | GCCGATCGGTCGATTCGC | GCCGATCGGTCGATTCGT | CACTACGCAACGCTTGTCC | AGCGGTGGCGGAACA |
| GFFK01006592.1 | 753 | C | T | CGAGAAATTAGTTCAGCGGACC | GCGAGAAATTAGTTCAGCGGACT | CGGCCTTCGACCTCTTCTTC | TTCGAACTCGCTTGTTTGATGAA |
| GFFK01006724.1 | 2178 | G | C | TCCTGATTGAGGATAACGCTTCG | TCCTGATTGAGGATAACGCTTCC | CTGGATCTGTACGATTCCATCATCTCT | GGCGTTTTTCCTGATTGAGG |
| GFFK01006981.1 | 1047 | C | T | GCACTGAAGATGAAATGGTGGTTTTC | GCACTGAAGATGAAATGGTGGTTTTT | GTGCTTGGAGCTGATAGGC | CACTTTTATCGGGAGCAGTAGATTG |
| GFFK01007257.1 | 900 | C | G | CTGAAGAAGCGAACAATGCCC | CTGAAGAAGCGAACAATGCCG | GGAACAGGAATTTTCGAGTCCTTTGA | CGTGGTAATCATCCTAAAATGGGAA |
| GFFK01008331.1 | 681 | A | C | GCGTAGTTGAACATTTCGCGT | GCGTAGTTGAACATTTCGCGG | GGCGAATCGGCCAGATTGA | ATCCATGAAGATGATACACGGTTG |
| GFFK01009096.1 | 183 | A | G | AGTAGAAAACTGTTTGTCGGAGGTTTA | GTAGAAAACTGTTTGTCGGAGGTTTG | GCGTAACTTTTCCCCAGCGG | GTCTCCGGATTTTGATGATGACA |
| GFFK01009187.1 | 837 | C | T | CCACCAGGCGCATGTTC | CCACCAGGCGCATGTTT | GTTGCCAACTGCCAGATTGGA | CCCGCGTGTGAAGAATATCA |
| GFFK01009819.1 | 1020 | A | G | CTCCAGGTGAGTGCCCA | CTCCAGGTGAGTGCCCG | GGATGGCAGAAGCGCCG | CCTACTTGTCTGGGTACTATCTGT |
| GFFK01012002.1 | 402 | C | T | AGGACATTTGTTTGTACGCTTGC | GAGGACATTTGTTTGTACGCTTGT | CAACGCTTCACCGTGATCGAA | GCGTTTCATAGTTTCCCGACC |

# *F*_ST_ Analyes

*F*_ST_ values were calculated for both SNP data and NADH5 using Arlequin 3.5.2.2 (Excoffier & Lischer, 2010) and the Empirical Bayes method (EB*F*_ST_) in FinePop 1.5.1 (Kitada, Nakamichi, & Kishino, 2017). We tested our SNP data for deviation from Hardy-Weinberg equilibrium and linkage disequilibrium. Significant deviations from the Hardy-Weinberg equilibrium (Pegas package in R, Paradis (2010)) and significant linkage disequilibrium (tested in Arlequin 3.5.2.2 with default settings) where only found for loci with low MAF. In these loci, particular genotypes were absent from populations or the entire dataset. The significant results were therefore likely to be spurious and we did not reduce the number of loci based on these tests. EB*F*_ST_ was calculated for mitochondrial data in the form of a haplotype frequency table in Finepop (Kitada et al., 2017), while sequences with their frequency per population were entered for Arlequin 3.5.2.2. As described in the main text, Mitochondrial sequences were highly variable and thus simplified to groups of nearly identical sequences for *F*_ST_ analyses. This assignment is visualised in Fig. S3. Analysis specifications are provided in Table S6 and visualisation of pairwise EB*F*_ST_ is shown in Fig. S4 with neighbour joining trees.


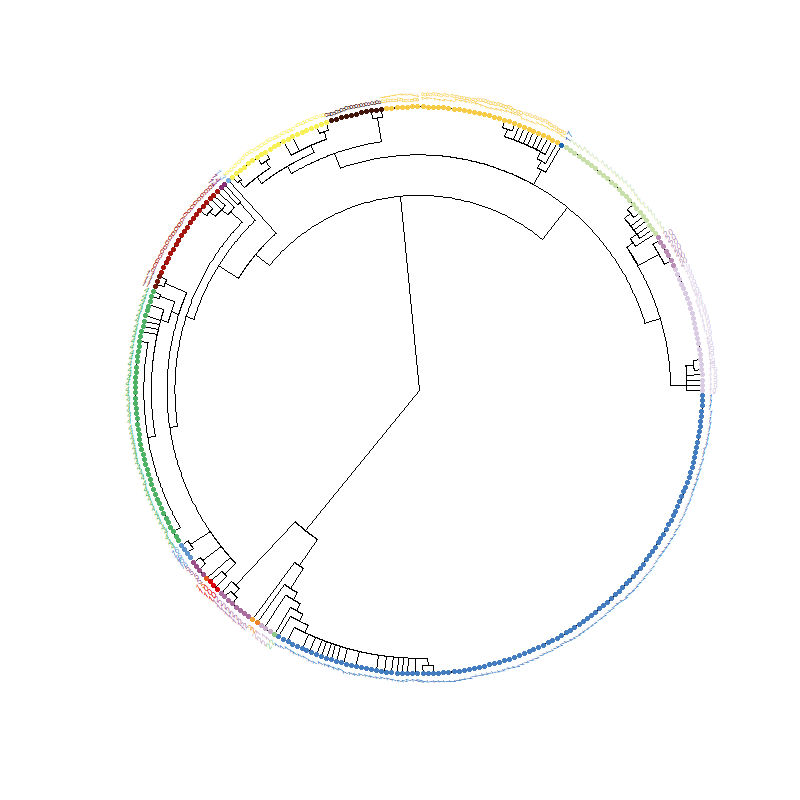


### **Fig. S3.** Haplotype assignment for *F*_ST_ analysis.

All 330 *A. winterbourni* sequences from the lakes that were included in *F*_ST_ analysis are placed in 21 groups of highly similar sequences based on genetic distance.

### **Table S4.1.** Pairwise *F*_ST_ tables using Arlequin.

The lower triangle shows pairwise *F*_ST_ values, while upper triangle indicates significance (**p* < 0.05).

| **Arlequin *F*_ST_ SNP data** | | Mackenzie District | | | Ashburton lakes | |  | Craigieburn Range | | | West coast lakes (north to south) | | | | | |
| --- | --- | --- | --- | --- | --- | --- | --- | --- | --- | --- | --- | --- | --- | --- | --- | --- |
|  |  | Alexandrina | Benmore | Middleton | Emma | Clearwater | Ellesmere | Selfe | Pearson | Grasmere | Haupiri | Kaniere | Mapourika | Paringa | Moeraki | Ellery |
| Mackenzie District | Alexandrina |  | - | * | - | - | - | * | * | - | * | * | * | * | * | * |
|  | Benmore | 0.007 |  | - | - | - | - | - | - | - | * | - | * | - | * | - |
|  | Middleton | 0.022 | 0.009 |  | - | - | - | - | * | - | * | * | * | * | * | * |
| Ashburton lakes | Emma | 0.002 | -0.003 | 0.011 |  | - | - | - | - | - | * | - | * | - | - | - |
|  | Clearwater | 0.001 | 0.010 | 0.005 | -0.005 |  | - | - | - | - | * | - | - | - | - | - |
|  | Ellesmere | 0.017 | -0.004 | -0.029 | 0.003 | -0.029 |  | - | - | - | - | - | - | - | - | - |
| Craigieburn Range | Selfe | 0.008 | 0.002 | 0.006 | 0.009 | 0.003 | -0.014 |  | - | - | * | * | * | * | * | * |
|  | Pearson | 0.025 | 0.014 | 0.016 | 0.013 | 0.003 | -0.003 | 0.008 |  | - | - | - | * | - | * | - |
|  | Grasmere | 0.012 | 0.001 | 0.014 | 0.009 | 0.009 | -0.002 | -0.001 | -0.013 |  | - | - | - | - | - | - |
| West coast lakes (north to south) | Haupiri | 0.052 | 0.029 | 0.046 | 0.050 | 0.038 | 0.026 | 0.027 | 0.005 | -0.013 |  | - | - | * | * | * |
|  | Kaniere | 0.036 | 0.020 | 0.035 | 0.004 | 0.011 | 0.018 | 0.030 | 0.017 | 0.002 | 0.016 |  | - | - | - | - |
|  | Mapourika | 0.045 | 0.027 | 0.035 | 0.033 | 0.026 | 0.007 | 0.028 | 0.015 | 0.000 | 0.010 | 0.008 |  | * | * | - |
|  | Paringa | 0.018 | 0.010 | 0.035 | 0.009 | 0.013 | 0.016 | 0.009 | 0.007 | -0.001 | 0.016 | 0.007 | 0.020 |  | * | - |
|  | Moeraki | 0.034 | 0.012 | 0.024 | 0.015 | 0.016 | 0.010 | 0.021 | 0.013 | -0.008 | 0.012 | -0.006 | 0.013 | 0.010 |  | - |
|  | Ellery | 0.043 | 0.035 | 0.057 | 0.019 | -0.003 | 0.040 | 0.041 | 0.025 | 0.023 | 0.035 | 0.036 | 0.021 | 0.012 | 0.025 |  |

| **Arlequin *F*_ST_ mitochondrial** | | Mackenzie District | | | Ashburton lakes | |  | Craigieburn Range | | | West coast lakes (north to south) | | | | | |
| --- | --- | --- | --- | --- | --- | --- | --- | --- | --- | --- | --- | --- | --- | --- | --- | --- |
|  |  | Alexandrina | Benmore | Middleton | Emma | Clearwater | Ellesmere | Selfe | Pearson | Grasmere | Haupiri | Kaniere | Mapourika | Paringa | Moeraki | Ellery |
| Mackenzie District | Alexandrina |  | * | - | * | * | * | * | * | * | * | * | * | * | * | * |
|  | Benmore | 0.126 |  | - | * | * | * | * | * | * | * | * | * | * | * | * |
|  | Middleton | 0.042 | -0.013 |  | * | * | * | * | * | * | * | * | * | * | * | * |
| Ashburton lakes | Emma | 0.975 | 0.837 | 0.876 |  | - | * | - | - | - | * | * | * | * | * | * |
|  | Clearwater | 0.959 | 0.764 | 0.816 | -0.069 |  | * | - | - | - | * | * | * | * | * | - |
|  | Ellesmere | 0.965 | 0.686 | 0.762 | 0.908 | 0.797 |  | * | * | * | * | * | * | * | * | * |
| Craigieburn Range | Selfe | 0.979 | 0.906 | 0.925 | 0.118 | 0.158 | 0.959 |  | - | - | * | * | * | * | * | * |
|  | Pearson | 0.973 | 0.892 | 0.911 | 0.067 | 0.137 | 0.939 | 0.023 |  | - | * | * | * | * | * | * |
|  | Grasmere | 0.980 | 0.869 | 0.901 | 0.009 | 0.056 | 0.947 | 0.038 | -0.024 |  | * | * | * | * | * | * |
| West coast lakes (north to south) | Haupiri | 0.967 | 0.852 | 0.879 | 0.371 | 0.318 | 0.890 | 0.576 | 0.511 | 0.437 |  | - | * | * | * | - |
|  | Kaniere | 0.980 | 0.862 | 0.894 | 0.516 | 0.407 | 0.930 | 0.746 | 0.673 | 0.615 | -0.020 |  | - | - | * | - |
|  | Mapourika | 0.975 | 0.900 | 0.917 | 0.706 | 0.669 | 0.943 | 0.811 | 0.767 | 0.750 | 0.330 | 0.218 |  | * | * | * |
|  | Paringa | 0.970 | 0.897 | 0.912 | 0.691 | 0.657 | 0.929 | 0.788 | 0.751 | 0.728 | 0.222 | 0.046 | 0.357 |  | * | - |
|  | Moeraki | 0.968 | 0.905 | 0.917 | 0.718 | 0.697 | 0.934 | 0.797 | 0.764 | 0.747 | 0.355 | 0.239 | 0.343 | 0.202 |  | - |
|  | Ellery | 0.980 | 0.852 | 0.888 | 0.460 | 0.339 | 0.921 | 0.726 | 0.650 | 0.574 | 0.027 | -0.052 | 0.342 | 0.006 | 0.145 |  |

### **Table S4.2.** Pairwise *F*_ST_ tables using EB*F*_ST_.

| **EB *F*_ST_ SNP data** | | Mackenzie District | | | Ashburton lakes | |  | Craigieburn Range | | | West coast lakes (north to south) | | | | | |
| --- | --- | --- | --- | --- | --- | --- | --- | --- | --- | --- | --- | --- | --- | --- | --- | --- |
|  |  | Alexandrina | Benmore | Middleton | Emma | Clearwater | Ellesmere | Selfe | Pearson | Grasmere | Haupiri | Kaniere | Mapourika | Paringa | Moeraki | Ellery |
| Mackenzie District | Alexandrina | 0.000 | 0.008 | 0.010 | 0.009 | 0.009 | 0.010 | 0.007 | 0.011 | 0.010 | 0.013 | 0.011 | 0.013 | 0.009 | 0.012 | 0.011 |
|  | Benmore | 0.008 | 0.000 | 0.007 | 0.007 | 0.008 | 0.008 | 0.007 | 0.008 | 0.008 | 0.009 | 0.008 | 0.009 | 0.008 | 0.008 | 0.008 |
|  | Middleton | 0.010 | 0.007 | 0.000 | 0.008 | 0.008 | 0.007 | 0.007 | 0.008 | 0.008 | 0.010 | 0.009 | 0.010 | 0.010 | 0.009 | 0.009 |
| Ashburton lakes | Emma | 0.009 | 0.007 | 0.008 | 0.000 | 0.008 | 0.008 | 0.008 | 0.008 | 0.008 | 0.010 | 0.008 | 0.009 | 0.008 | 0.008 | 0.008 |
|  | Clearwater | 0.009 | 0.008 | 0.008 | 0.008 | 0.000 | 0.007 | 0.008 | 0.008 | 0.008 | 0.009 | 0.008 | 0.009 | 0.008 | 0.008 | 0.008 |
|  | Ellesmere | 0.010 | 0.008 | 0.007 | 0.008 | 0.007 | 0.000 | 0.007 | 0.007 | 0.008 | 0.009 | 0.008 | 0.008 | 0.008 | 0.008 | 0.008 |
| Craigieburn Range | Selfe | 0.007 | 0.007 | 0.007 | 0.008 | 0.008 | 0.007 | 0.000 | 0.007 | 0.007 | 0.009 | 0.009 | 0.009 | 0.007 | 0.009 | 0.009 |
|  | Pearson | 0.011 | 0.008 | 0.008 | 0.008 | 0.008 | 0.007 | 0.007 | 0.000 | 0.006 | 0.007 | 0.008 | 0.008 | 0.007 | 0.008 | 0.008 |
|  | Grasmere | 0.010 | 0.008 | 0.008 | 0.008 | 0.008 | 0.008 | 0.007 | 0.006 | 0.000 | 0.007 | 0.008 | 0.008 | 0.007 | 0.007 | 0.008 |
| West coast lakes (north to south) | Haupiri | 0.013 | 0.009 | 0.010 | 0.010 | 0.009 | 0.009 | 0.009 | 0.007 | 0.007 | 0.000 | 0.008 | 0.008 | 0.008 | 0.007 | 0.009 |
|  | Kaniere | 0.011 | 0.008 | 0.009 | 0.008 | 0.008 | 0.008 | 0.009 | 0.008 | 0.008 | 0.008 | 0.000 | 0.008 | 0.007 | 0.006 | 0.008 |
|  | Mapourika | 0.013 | 0.009 | 0.010 | 0.009 | 0.009 | 0.008 | 0.009 | 0.008 | 0.008 | 0.008 | 0.008 | 0.000 | 0.008 | 0.007 | 0.008 |
|  | Paringa | 0.009 | 0.008 | 0.010 | 0.008 | 0.008 | 0.008 | 0.007 | 0.007 | 0.007 | 0.008 | 0.007 | 0.008 | 0.000 | 0.007 | 0.008 |
|  | Moeraki | 0.012 | 0.008 | 0.009 | 0.008 | 0.008 | 0.008 | 0.009 | 0.008 | 0.007 | 0.007 | 0.006 | 0.007 | 0.007 | 0.000 | 0.008 |
|  | Ellery | 0.011 | 0.008 | 0.009 | 0.008 | 0.008 | 0.008 | 0.009 | 0.008 | 0.008 | 0.009 | 0.008 | 0.008 | 0.008 | 0.008 | 0.000 |

| **EB *F*_ST_ mitochondrial** | | Mackenzie District | | | Ashburton lakes | |  | Craigieburn Range | | | West coast lakes (north to south) | | | | | |
| --- | --- | --- | --- | --- | --- | --- | --- | --- | --- | --- | --- | --- | --- | --- | --- | --- |
|  |  | Alexandrina | Benmore | Middleton | Emma | Clearwater | Ellesmere | Selfe | Pearson | Grasmere | Haupiri | Kaniere | Mapourika | Paringa | Moeraki | Ellery |
| Mackenzie District | Alexandrina | 0.000 | 0.095 | 0.040 | 0.426 | 0.515 | 0.707 | 0.550 | 0.458 | 0.427 | 0.504 | 0.458 | 0.461 | 0.428 | 0.492 | 0.470 |
|  | Benmore | 0.095 | 0.000 | 0.053 | 0.260 | 0.323 | 0.483 | 0.351 | 0.282 | 0.261 | 0.322 | 0.290 | 0.296 | 0.271 | 0.321 | 0.298 |
|  | Middleton | 0.040 | 0.053 | 0.000 | 0.340 | 0.417 | 0.584 | 0.449 | 0.368 | 0.341 | 0.399 | 0.360 | 0.368 | 0.340 | 0.399 | 0.378 |
| Ashburton lakes | Emma | 0.426 | 0.260 | 0.340 | 0.000 | 0.091 | 0.324 | 0.097 | 0.072 | 0.081 | 0.149 | 0.152 | 0.173 | 0.151 | 0.192 | 0.155 |
|  | Clearwater | 0.515 | 0.323 | 0.417 | 0.091 | 0.000 | 0.347 | 0.099 | 0.077 | 0.109 | 0.174 | 0.196 | 0.232 | 0.207 | 0.253 | 0.196 |
|  | Ellesmere | 0.707 | 0.483 | 0.584 | 0.324 | 0.347 | 0.000 | 0.423 | 0.347 | 0.324 | 0.387 | 0.352 | 0.354 | 0.324 | 0.379 | 0.363 |
| Craigieburn Range | Selfe | 0.550 | 0.351 | 0.449 | 0.097 | 0.099 | 0.423 | 0.000 | 0.038 | 0.070 | 0.192 | 0.216 | 0.251 | 0.228 | 0.275 | 0.215 |
|  | Pearson | 0.458 | 0.282 | 0.368 | 0.072 | 0.077 | 0.347 | 0.038 | 0.000 | 0.044 | 0.147 | 0.163 | 0.192 | 0.170 | 0.212 | 0.163 |
|  | Grasmere | 0.427 | 0.261 | 0.341 | 0.081 | 0.109 | 0.324 | 0.070 | 0.044 | 0.000 | 0.150 | 0.152 | 0.173 | 0.152 | 0.192 | 0.155 |
| West coast lakes (north to south) | Haupiri | 0.504 | 0.322 | 0.399 | 0.149 | 0.174 | 0.387 | 0.192 | 0.147 | 0.150 | 0.000 | 0.060 | 0.154 | 0.158 | 0.229 | 0.206 |
|  | Kaniere | 0.458 | 0.290 | 0.360 | 0.152 | 0.196 | 0.352 | 0.216 | 0.163 | 0.152 | 0.060 | 0.000 | 0.119 | 0.113 | 0.183 | 0.170 |
|  | Mapourika | 0.461 | 0.296 | 0.368 | 0.173 | 0.232 | 0.354 | 0.251 | 0.192 | 0.173 | 0.154 | 0.119 | 0.000 | 0.150 | 0.189 | 0.204 |
|  | Paringa | 0.428 | 0.271 | 0.340 | 0.151 | 0.207 | 0.324 | 0.228 | 0.170 | 0.152 | 0.158 | 0.113 | 0.150 | 0.000 | 0.072 | 0.061 |
|  | Moeraki | 0.492 | 0.321 | 0.399 | 0.192 | 0.253 | 0.379 | 0.275 | 0.212 | 0.192 | 0.229 | 0.183 | 0.189 | 0.072 | 0.000 | 0.101 |
|  | Ellery | 0.470 | 0.298 | 0.378 | 0.155 | 0.196 | 0.363 | 0.215 | 0.163 | 0.155 | 0.206 | 0.170 | 0.204 | 0.061 | 0.101 | 0.000 |


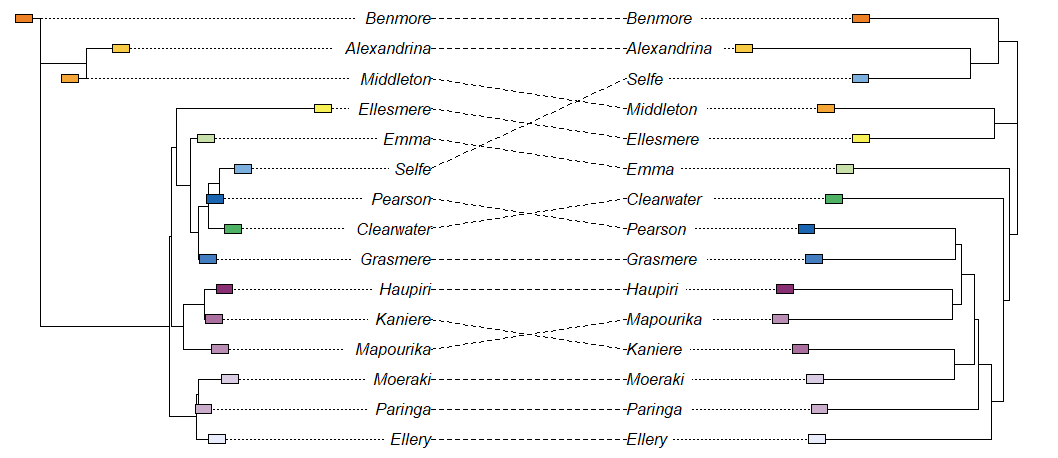


### **Fig. S4.** Neighbour-Joining trees showing mito-nuclear congruence.

Mitochondrial (left) and nuclear genes (right) in pairwise EB*F*_ST_ estimates, displayed with. Mitochondrial data shows better clustering of Lakes that are geographically close, but both methods support a differentiation of lakes South of Ashburton (Lake Alexandrina, Lake Benmore and Lake Middleton). In the nuclear genome Lake Selfe and Lake Ellesmere Cluster with the more Southern Lakes.

# Geographic distance matrices


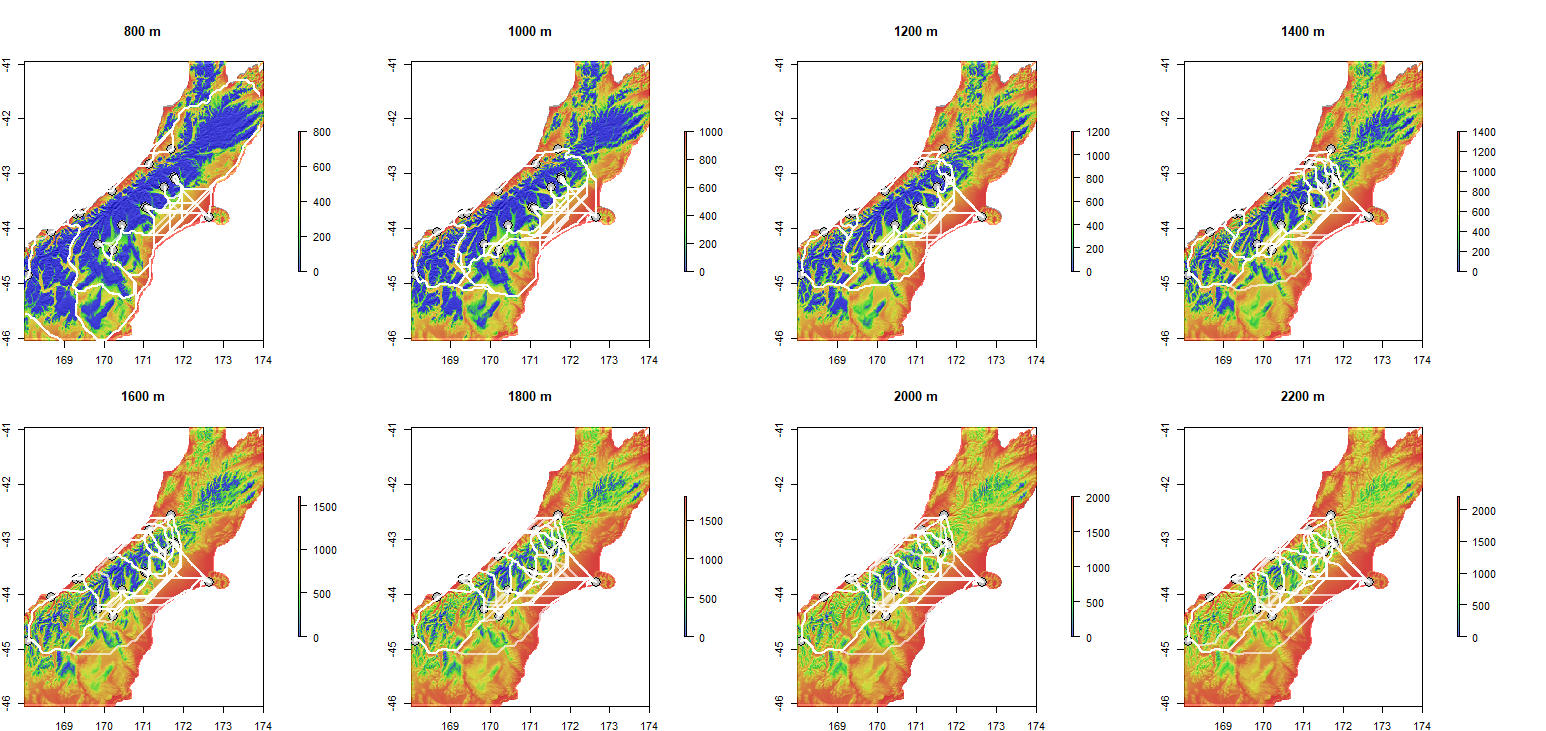


### **Fig. S5.** Topographic path analysis.

Distances between lakes in New Zealand were calculated using different maximum migration altitudes. Since isolation by distance was not significant for nuclear SNP data, we resorted to topographic path modelling to create alternative distance matrices between populations (Wang, 2020). These may be more biologically relevant than direct distances, which ignore geographical boundaries to gene flow.

### **Table S5.1.** Geographic distance matrix at a maximum migration altitude of 800 m

|  | Kaniere | Mapourika | Alexandrina | Grasmere | Pearson | Ellery | Clearwater | Ellesmere | Emma | Gunn | Middleton | Moeraki | Paringa | Selfe | Haupiri | Benmore |
| --- | --- | --- | --- | --- | --- | --- | --- | --- | --- | --- | --- | --- | --- | --- | --- | --- |
| Kaniere | 0.00 | 138.17 | 1631.76 | 1189.60 | 1174.81 | 339.73 | 1312.11 | 1038.54 | 1267.62 | 554.79 | 1381.03 | 259.21 | 242.08 | 1220.19 | 98.74 | 1283.14 |
| Mapourika | 138.17 | 0.00 | 1515.31 | 1302.42 | 1287.63 | 211.09 | 1351.59 | 1151.36 | 1305.20 | 426.15 | 1252.39 | 130.57 | 113.44 | 1333.01 | 224.58 | 1154.51 |
| Alexandrina | 1631.76 | 1515.31 | 0.00 | 793.31 | 778.52 | 1399.42 | 676.98 | 633.79 | 630.59 | 1263.80 | 427.00 | 1390.96 | 1407.40 | 705.96 | 1576.12 | 360.81 |
| Grasmere | 1189.60 | 1302.42 | 793.31 | 0.00 | 24.04 | 1327.81 | 449.47 | 260.36 | 404.98 | 1186.08 | 707.54 | 1319.35 | 1335.79 | 331.68 | 1133.97 | 609.65 |
| Pearson | 1174.81 | 1287.63 | 778.52 | 24.04 | 0.00 | 1313.02 | 434.67 | 245.57 | 390.19 | 1171.29 | 692.74 | 1304.56 | 1321.00 | 316.89 | 1119.18 | 594.86 |
| Ellery | 339.73 | 211.09 | 1399.42 | 1327.81 | 1313.02 | 0.00 | 1235.69 | 1161.84 | 1189.31 | 217.98 | 1136.50 | 87.28 | 103.18 | 1248.90 | 426.13 | 1038.62 |
| Clearwater | 1312.11 | 1351.59 | 676.98 | 449.47 | 434.67 | 1235.69 | 0.00 | 324.00 | 48.99 | 1093.97 | 615.42 | 1227.23 | 1243.67 | 339.12 | 1256.48 | 517.54 |
| Ellesmere | 1038.54 | 1151.36 | 633.79 | 260.36 | 245.57 | 1161.84 | 324.00 | 0.00 | 279.52 | 1020.11 | 541.56 | 1153.37 | 1169.82 | 248.61 | 982.91 | 443.68 |
| Emma | 1267.62 | 1305.20 | 630.59 | 404.98 | 390.19 | 1189.31 | 48.99 | 279.52 | 0.00 | 1047.58 | 569.03 | 1180.85 | 1197.29 | 294.63 | 1211.99 | 471.15 |
| Gunn | 554.79 | 426.15 | 1263.80 | 1186.08 | 1171.29 | 217.98 | 1093.97 | 1020.11 | 1047.58 | 0.00 | 1000.88 | 302.34 | 318.24 | 1107.18 | 641.19 | 902.99 |
| Middleton | 1381.03 | 1252.39 | 427.00 | 707.54 | 692.74 | 1136.50 | 615.42 | 541.56 | 569.03 | 1000.88 | 0.00 | 1128.04 | 1144.48 | 628.63 | 1467.44 | 111.45 |
| Moeraki | 259.21 | 130.57 | 1390.96 | 1319.35 | 1304.56 | 87.28 | 1227.23 | 1153.37 | 1180.85 | 302.34 | 1128.04 | 0.00 | 19.34 | 1240.44 | 345.62 | 1030.16 |
| Paringa | 242.08 | 113.44 | 1407.40 | 1335.79 | 1321.00 | 103.18 | 1243.67 | 1169.82 | 1197.29 | 318.24 | 1144.48 | 19.34 | 0.00 | 1256.88 | 328.48 | 1046.60 |
| Selfe | 1220.19 | 1333.01 | 705.96 | 331.68 | 316.89 | 1248.90 | 339.12 | 248.61 | 294.63 | 1107.18 | 628.63 | 1240.44 | 1256.88 | 0.00 | 1164.56 | 530.75 |
| Haupiri | 98.74 | 224.58 | 1576.12 | 1133.97 | 1119.18 | 426.13 | 1256.48 | 982.91 | 1211.99 | 641.19 | 1467.44 | 345.62 | 328.48 | 1164.56 | 0.00 | 1369.55 |
| Benmore | 1283.14 | 1154.51 | 360.81 | 609.65 | 594.86 | 1038.62 | 517.54 | 443.68 | 471.15 | 902.99 | 111.45 | 1030.16 | 1046.60 | 530.75 | 1369.55 | 0.00 |

### **Table S5.2.** Geographic distance matrix at a maximum migration altitude of 1000 m

|  | Kaniere | Mapourika | Alexandrina | Grasmere | Pearson | Ellery | Clearwater | Ellesmere | Emma | Gunn | Middleton | Moeraki | Paringa | Selfe | Haupiri | Benmore |
| --- | --- | --- | --- | --- | --- | --- | --- | --- | --- | --- | --- | --- | --- | --- | --- | --- |
| Kaniere | 0.00 | 108.09 | 746.84 | 527.38 | 518.13 | 268.66 | 617.94 | 469.09 | 599.75 | 422.50 | 700.09 | 203.85 | 191.07 | 566.90 | 74.63 | 720.06 |
| Mapourika | 108.09 | 0.00 | 734.83 | 627.19 | 617.95 | 166.48 | 717.75 | 568.91 | 699.57 | 320.32 | 597.91 | 101.68 | 88.90 | 666.72 | 174.44 | 617.89 |
| Alexandrina | 746.84 | 734.83 | 0.00 | 397.30 | 388.05 | 644.60 | 286.49 | 315.09 | 269.23 | 676.84 | 171.39 | 636.99 | 649.68 | 339.92 | 672.35 | 136.46 |
| Grasmere | 527.38 | 627.19 | 397.30 | 0.00 | 11.47 | 787.77 | 258.77 | 167.94 | 240.58 | 841.44 | 448.37 | 722.96 | 710.18 | 192.04 | 452.89 | 404.49 |
| Pearson | 518.13 | 617.95 | 388.05 | 11.47 | 0.00 | 778.52 | 249.52 | 158.69 | 231.33 | 832.19 | 439.12 | 713.71 | 700.93 | 182.79 | 443.64 | 395.24 |
| Ellery | 268.66 | 166.48 | 644.60 | 787.77 | 778.52 | 0.00 | 786.96 | 729.48 | 769.70 | 154.02 | 507.68 | 69.08 | 81.34 | 827.29 | 335.02 | 527.66 |
| Clearwater | 617.94 | 717.75 | 286.49 | 258.77 | 249.52 | 786.96 | 0.00 | 202.28 | 18.75 | 749.25 | 341.15 | 779.35 | 792.03 | 185.59 | 543.45 | 297.27 |
| Ellesmere | 469.09 | 568.91 | 315.09 | 167.94 | 158.69 | 729.48 | 202.28 | 0.00 | 184.10 | 751.24 | 363.98 | 664.67 | 651.90 | 171.68 | 394.61 | 320.10 |
| Emma | 599.75 | 699.57 | 269.23 | 240.58 | 231.33 | 769.70 | 18.75 | 184.10 | 0.00 | 732.00 | 323.89 | 762.09 | 774.77 | 167.41 | 525.27 | 280.02 |
| Gunn | 422.50 | 320.32 | 676.84 | 841.44 | 832.19 | 154.02 | 749.25 | 751.24 | 732.00 | 0.00 | 539.92 | 222.92 | 235.18 | 791.56 | 488.85 | 559.90 |
| Middleton | 700.09 | 597.91 | 171.39 | 448.37 | 439.12 | 507.68 | 341.15 | 363.98 | 323.89 | 539.92 | 0.00 | 500.07 | 512.76 | 392.00 | 721.94 | 68.67 |
| Moeraki | 203.85 | 101.68 | 636.99 | 722.96 | 713.71 | 69.08 | 779.35 | 664.67 | 762.09 | 222.92 | 500.07 | 0.00 | 13.99 | 762.48 | 270.21 | 520.05 |
| Paringa | 191.07 | 88.90 | 649.68 | 710.18 | 700.93 | 81.34 | 792.03 | 651.90 | 774.77 | 235.18 | 512.76 | 13.99 | 0.00 | 749.70 | 257.43 | 532.74 |
| Selfe | 566.90 | 666.72 | 339.92 | 192.04 | 182.79 | 827.29 | 185.59 | 171.68 | 167.41 | 791.56 | 392.00 | 762.48 | 749.70 | 0.00 | 492.41 | 348.12 |
| Haupiri | 74.63 | 174.44 | 672.35 | 452.89 | 443.64 | 335.02 | 543.45 | 394.61 | 525.27 | 488.85 | 721.94 | 270.21 | 257.43 | 492.41 | 0.00 | 678.06 |
| Benmore | 720.06 | 617.89 | 136.46 | 404.49 | 395.24 | 527.66 | 297.27 | 320.10 | 280.02 | 559.90 | 68.67 | 520.05 | 532.74 | 348.12 | 678.06 | 0.00 |

### **Table S5.3.** Geographic distance matrix at a maximum migration altitude of 1200 m

|  | Kaniere | Mapourika | Alexandrina | Grasmere | Pearson | Ellery | Clearwater | Ellesmere | Emma | Gunn | Middleton | Moeraki | Paringa | Selfe | Haupiri | Benmore |
| --- | --- | --- | --- | --- | --- | --- | --- | --- | --- | --- | --- | --- | --- | --- | --- | --- |
| Kaniere | 0.00 | 88.71 | 415.88 | 150.34 | 157.87 | 222.17 | 317.64 | 259.49 | 306.27 | 342.53 | 460.01 | 167.97 | 157.80 | 267.67 | 60.06 | 428.29 |
| Mapourika | 88.71 | 0.00 | 477.65 | 235.96 | 243.50 | 137.49 | 403.26 | 345.12 | 391.89 | 257.85 | 385.51 | 83.29 | 73.12 | 353.29 | 142.73 | 404.49 |
| Alexandrina | 415.88 | 477.65 | 0.00 | 282.25 | 276.33 | 415.17 | 184.94 | 231.73 | 174.38 | 421.88 | 113.49 | 410.90 | 416.41 | 237.77 | 406.93 | 90.17 |
| Grasmere | 150.34 | 235.96 | 282.25 | 0.00 | 7.54 | 369.42 | 184.01 | 125.86 | 172.63 | 489.78 | 326.37 | 315.22 | 305.05 | 119.43 | 141.38 | 294.66 |
| Pearson | 157.87 | 243.50 | 276.33 | 7.54 | 0.00 | 376.96 | 178.09 | 119.94 | 166.72 | 497.32 | 320.46 | 322.75 | 312.59 | 111.89 | 148.92 | 288.74 |
| Ellery | 222.17 | 137.49 | 415.17 | 369.42 | 376.96 | 0.00 | 536.73 | 478.58 | 525.35 | 120.48 | 323.03 | 57.18 | 67.16 | 486.75 | 276.19 | 342.01 |
| Clearwater | 317.64 | 403.26 | 184.94 | 184.01 | 178.09 | 536.73 | 0.00 | 152.81 | 11.60 | 522.89 | 238.39 | 482.52 | 472.35 | 129.55 | 308.69 | 207.49 |
| Ellesmere | 259.49 | 345.12 | 231.73 | 125.86 | 119.94 | 478.58 | 152.81 | 0.00 | 141.44 | 538.97 | 273.41 | 424.37 | 414.21 | 133.28 | 250.54 | 241.70 |
| Emma | 306.27 | 391.89 | 174.38 | 172.63 | 166.72 | 525.35 | 11.60 | 141.44 | 0.00 | 512.29 | 227.83 | 471.15 | 460.98 | 118.17 | 297.31 | 196.93 |
| Gunn | 342.53 | 257.85 | 421.88 | 489.78 | 497.32 | 120.48 | 522.89 | 538.97 | 512.29 | 0.00 | 329.74 | 177.54 | 187.51 | 561.48 | 396.55 | 348.71 |
| Middleton | 460.01 | 385.51 | 113.49 | 326.37 | 320.46 | 323.03 | 238.39 | 273.41 | 227.83 | 329.74 | 0.00 | 318.76 | 324.27 | 283.88 | 451.05 | 49.64 |
| Moeraki | 167.97 | 83.29 | 410.90 | 315.22 | 322.75 | 57.18 | 482.52 | 424.37 | 471.15 | 177.54 | 318.76 | 0.00 | 10.81 | 432.55 | 221.98 | 337.74 |
| Paringa | 157.80 | 73.12 | 416.41 | 305.05 | 312.59 | 67.16 | 472.35 | 414.21 | 460.98 | 187.51 | 324.27 | 10.81 | 0.00 | 422.38 | 211.82 | 343.25 |
| Selfe | 267.67 | 353.29 | 237.77 | 119.43 | 111.89 | 486.75 | 129.55 | 133.28 | 118.17 | 561.48 | 283.88 | 432.55 | 422.38 | 0.00 | 258.71 | 252.16 |
| Haupiri | 60.06 | 142.73 | 406.93 | 141.38 | 148.92 | 276.19 | 308.69 | 250.54 | 297.31 | 396.55 | 451.05 | 221.98 | 211.82 | 258.71 | 0.00 | 419.34 |
| Benmore | 428.29 | 404.49 | 90.17 | 294.66 | 288.74 | 342.01 | 207.49 | 241.70 | 196.93 | 348.71 | 49.64 | 337.74 | 343.25 | 252.16 | 419.34 | 0.00 |

### **Table S5.4.** Geographic distance matrix at a maximum migration altitude of 1400 m

|  | Kaniere | Mapourika | Alexandrina | Grasmere | Pearson | Ellery | Clearwater | Ellesmere | Emma | Gunn | Middleton | Moeraki | Paringa | Selfe | Haupiri | Benmore |
| --- | --- | --- | --- | --- | --- | --- | --- | --- | --- | --- | --- | --- | --- | --- | --- | --- |
| Kaniere | 0.00 | 75.20 | 302.57 | 103.75 | 109.36 | 189.43 | 183.53 | 200.04 | 179.95 | 288.57 | 343.15 | 142.70 | 134.42 | 124.14 | 50.21 | 317.98 |
| Mapourika | 75.20 | 0.00 | 350.89 | 176.91 | 182.53 | 117.12 | 233.63 | 272.92 | 230.05 | 216.26 | 281.22 | 70.39 | 62.12 | 195.14 | 120.77 | 297.89 |
| Alexandrina | 302.57 | 350.89 | 0.00 | 219.82 | 215.44 | 293.11 | 133.45 | 185.73 | 126.04 | 315.34 | 83.60 | 298.10 | 298.74 | 184.04 | 305.98 | 67.99 |
| Grasmere | 103.75 | 176.91 | 219.82 | 0.00 | 5.61 | 291.15 | 143.23 | 101.49 | 134.96 | 390.28 | 258.83 | 244.42 | 236.14 | 74.97 | 95.26 | 233.66 |
| Pearson | 109.36 | 182.53 | 215.44 | 5.61 | 0.00 | 296.76 | 138.86 | 97.14 | 130.58 | 395.89 | 254.46 | 250.03 | 241.75 | 69.50 | 100.87 | 229.29 |
| Ellery | 189.43 | 117.12 | 293.11 | 291.15 | 296.76 | 0.00 | 348.76 | 387.15 | 345.19 | 99.22 | 223.45 | 48.78 | 57.14 | 309.37 | 235.00 | 240.12 |
| Clearwater | 183.53 | 233.63 | 133.45 | 143.23 | 138.86 | 348.76 | 0.00 | 123.93 | 8.40 | 408.25 | 178.47 | 302.04 | 293.76 | 98.89 | 229.40 | 154.57 |
| Ellesmere | 200.04 | 272.92 | 185.73 | 101.49 | 97.14 | 387.15 | 123.93 | 0.00 | 115.65 | 429.29 | 222.19 | 340.43 | 332.15 | 109.57 | 187.65 | 197.02 |
| Emma | 179.95 | 230.05 | 126.04 | 134.96 | 130.58 | 345.19 | 8.40 | 115.65 | 0.00 | 400.84 | 171.06 | 298.46 | 290.18 | 91.24 | 221.12 | 147.16 |
| Gunn | 288.57 | 216.26 | 315.34 | 390.28 | 395.89 | 99.22 | 408.25 | 429.29 | 400.84 | 0.00 | 245.68 | 147.92 | 156.28 | 408.51 | 334.13 | 262.35 |
| Middleton | 343.15 | 281.22 | 83.60 | 258.83 | 254.46 | 223.45 | 178.47 | 222.19 | 171.06 | 245.68 | 0.00 | 228.43 | 229.08 | 224.62 | 345.00 | 38.91 |
| Moeraki | 142.70 | 70.39 | 298.10 | 244.42 | 250.03 | 48.78 | 302.04 | 340.43 | 298.46 | 147.92 | 228.43 | 0.00 | 8.76 | 262.64 | 188.27 | 245.10 |
| Paringa | 134.42 | 62.12 | 298.74 | 236.14 | 241.75 | 57.14 | 293.76 | 332.15 | 290.18 | 156.28 | 229.08 | 8.76 | 0.00 | 254.36 | 179.99 | 245.74 |
| Selfe | 124.14 | 195.14 | 184.04 | 74.97 | 69.50 | 309.37 | 98.89 | 109.57 | 91.24 | 408.51 | 224.62 | 262.64 | 254.36 | 0.00 | 149.08 | 199.45 |
| Haupiri | 50.21 | 120.77 | 305.98 | 95.26 | 100.87 | 235.00 | 229.40 | 187.65 | 221.12 | 334.13 | 345.00 | 188.27 | 179.99 | 149.08 | 0.00 | 319.83 |
| Benmore | 317.98 | 297.89 | 67.99 | 233.66 | 229.29 | 240.12 | 154.57 | 197.02 | 147.16 | 262.35 | 38.91 | 245.10 | 245.74 | 199.45 | 319.83 | 0.00 |

### **Table S5.5.** Geographic distance matrix at a maximum migration altitude of 1600 m

|  | Kaniere | Mapourika | Alexandrina | Grasmere | Pearson | Ellery | Clearwater | Ellesmere | Emma | Gunn | Middleton | Moeraki | Paringa | Selfe | Haupiri | Benmore |
| --- | --- | --- | --- | --- | --- | --- | --- | --- | --- | --- | --- | --- | --- | --- | --- | --- |
| Kaniere | 0.00 | 65.27 | 215.82 | 80.34 | 84.77 | 165.11 | 124.50 | 163.23 | 122.07 | 249.38 | 219.82 | 124.08 | 117.09 | 73.78 | 43.13 | 235.30 |
| Mapourika | 65.27 | 0.00 | 178.62 | 144.44 | 148.86 | 102.02 | 125.35 | 219.01 | 131.93 | 186.28 | 156.73 | 60.98 | 53.99 | 133.43 | 104.68 | 186.68 |
| Alexandrina | 215.82 | 178.62 | 0.00 | 180.16 | 176.37 | 199.37 | 100.78 | 155.74 | 95.08 | 253.24 | 66.24 | 178.66 | 171.63 | 150.17 | 246.50 | 54.72 |
| Grasmere | 80.34 | 144.44 | 180.16 | 0.00 | 4.47 | 244.28 | 113.76 | 85.30 | 107.26 | 328.55 | 215.01 | 203.25 | 196.26 | 48.23 | 70.27 | 194.18 |
| Pearson | 84.77 | 148.86 | 176.37 | 4.47 | 0.00 | 248.71 | 109.56 | 81.80 | 103.06 | 332.97 | 211.33 | 207.67 | 200.68 | 50.17 | 74.74 | 190.50 |
| Ellery | 165.11 | 102.02 | 199.37 | 244.28 | 248.71 | 0.00 | 227.34 | 319.61 | 233.92 | 84.33 | 139.01 | 42.54 | 49.68 | 233.28 | 204.53 | 168.95 |
| Clearwater | 124.50 | 125.35 | 100.78 | 113.76 | 109.56 | 227.34 | 0.00 | 104.58 | 6.58 | 311.61 | 143.04 | 186.31 | 179.32 | 77.91 | 166.75 | 123.55 |
| Ellesmere | 163.23 | 219.01 | 155.74 | 85.30 | 81.80 | 319.61 | 104.58 | 0.00 | 98.08 | 358.82 | 187.90 | 278.58 | 271.59 | 93.23 | 151.64 | 167.06 |
| Emma | 122.07 | 131.93 | 95.08 | 107.26 | 103.06 | 233.92 | 6.58 | 98.08 | 0.00 | 318.19 | 137.34 | 192.89 | 185.90 | 74.25 | 164.32 | 117.85 |
| Gunn | 249.38 | 186.28 | 253.24 | 328.55 | 332.97 | 84.33 | 311.61 | 358.82 | 318.19 | 0.00 | 197.13 | 126.80 | 133.95 | 317.55 | 288.79 | 211.65 |
| Middleton | 219.82 | 156.73 | 66.24 | 215.01 | 211.33 | 139.01 | 143.04 | 187.90 | 137.34 | 197.13 | 0.00 | 118.30 | 111.27 | 186.12 | 259.24 | 32.00 |
| Moeraki | 124.08 | 60.98 | 178.66 | 203.25 | 207.67 | 42.54 | 186.31 | 278.58 | 192.89 | 126.80 | 118.30 | 0.00 | 7.33 | 192.25 | 163.49 | 148.24 |
| Paringa | 117.09 | 53.99 | 171.63 | 196.26 | 200.68 | 49.68 | 179.32 | 271.59 | 185.90 | 133.95 | 111.27 | 7.33 | 0.00 | 185.26 | 156.50 | 141.22 |
| Selfe | 73.78 | 133.43 | 150.17 | 48.23 | 50.17 | 233.28 | 77.91 | 93.23 | 74.25 | 317.55 | 186.12 | 192.25 | 185.26 | 0.00 | 105.50 | 165.29 |
| Haupiri | 43.13 | 104.68 | 246.50 | 70.27 | 74.74 | 204.53 | 166.75 | 151.64 | 164.32 | 288.79 | 259.24 | 163.49 | 156.50 | 105.50 | 0.00 | 260.52 |
| Benmore | 235.30 | 186.68 | 54.72 | 194.18 | 190.50 | 168.95 | 123.55 | 167.06 | 117.85 | 211.65 | 32.00 | 148.24 | 141.22 | 165.29 | 260.52 | 0.00 |

### **Table S5.6.** Geographic distance matrix at a maximum migration altitude of 1800 m

|  | Kaniere | Mapourika | Alexandrina | Grasmere | Pearson | Ellery | Clearwater | Ellesmere | Emma | Gunn | Middleton | Moeraki | Paringa | Selfe | Haupiri | Benmore |
| --- | --- | --- | --- | --- | --- | --- | --- | --- | --- | --- | --- | --- | --- | --- | --- | --- |
| Kaniere | 0.00 | 57.66 | 152.50 | 65.91 | 69.40 | 146.34 | 96.32 | 133.68 | 94.56 | 219.42 | 175.54 | 109.77 | 103.72 | 55.27 | 37.80 | 191.62 |
| Mapourika | 57.66 | 0.00 | 113.67 | 121.03 | 124.52 | 90.37 | 85.39 | 174.82 | 90.80 | 163.45 | 119.57 | 53.81 | 47.75 | 107.96 | 92.39 | 135.88 |
| Alexandrina | 152.50 | 113.67 | 0.00 | 150.99 | 147.55 | 152.84 | 80.60 | 133.00 | 75.97 | 211.35 | 54.72 | 120.62 | 114.57 | 125.08 | 187.78 | 45.84 |
| Grasmere | 65.91 | 121.03 | 150.99 | 0.00 | 3.71 | 209.71 | 93.85 | 73.68 | 88.48 | 282.79 | 184.05 | 173.15 | 167.09 | 36.02 | 56.50 | 166.29 |
| Pearson | 69.40 | 124.52 | 147.55 | 3.71 | 0.00 | 213.19 | 90.44 | 70.67 | 85.07 | 286.28 | 180.42 | 176.63 | 170.58 | 38.73 | 60.21 | 162.66 |
| Ellery | 146.34 | 90.37 | 152.84 | 209.71 | 213.19 | 0.00 | 175.74 | 263.58 | 181.15 | 73.14 | 105.89 | 37.71 | 43.95 | 196.63 | 181.06 | 131.33 |
| Clearwater | 96.32 | 85.39 | 80.60 | 93.85 | 90.44 | 175.74 | 0.00 | 90.61 | 5.41 | 248.82 | 119.18 | 139.18 | 133.12 | 64.16 | 133.04 | 102.66 |
| Ellesmere | 133.68 | 174.82 | 133.00 | 73.68 | 70.67 | 263.58 | 90.61 | 0.00 | 85.25 | 308.82 | 163.04 | 228.60 | 222.55 | 80.85 | 126.55 | 145.28 |
| Emma | 94.56 | 90.80 | 75.97 | 88.48 | 85.07 | 181.15 | 5.41 | 85.25 | 0.00 | 254.23 | 114.55 | 144.59 | 138.53 | 62.40 | 131.28 | 98.03 |
| Gunn | 219.42 | 163.45 | 211.35 | 282.79 | 286.28 | 73.14 | 248.82 | 308.82 | 254.23 | 0.00 | 164.42 | 110.80 | 117.04 | 269.72 | 254.15 | 175.93 |
| Middleton | 175.54 | 119.57 | 54.72 | 184.05 | 180.42 | 105.89 | 119.18 | 163.04 | 114.55 | 164.42 | 0.00 | 84.73 | 79.31 | 158.81 | 210.27 | 27.18 |
| Moeraki | 109.77 | 53.81 | 120.62 | 173.15 | 176.63 | 37.71 | 139.18 | 228.60 | 144.59 | 110.80 | 84.73 | 0.00 | 6.30 | 160.07 | 144.50 | 110.17 |
| Paringa | 103.72 | 47.75 | 114.57 | 167.09 | 170.58 | 43.95 | 133.12 | 222.55 | 138.53 | 117.04 | 79.31 | 6.30 | 0.00 | 154.02 | 138.45 | 104.76 |
| Selfe | 55.27 | 107.96 | 125.08 | 36.02 | 38.73 | 196.63 | 64.16 | 80.85 | 62.40 | 269.72 | 158.81 | 160.07 | 154.02 | 0.00 | 83.15 | 141.06 |
| Haupiri | 37.80 | 92.39 | 187.78 | 56.50 | 60.21 | 181.06 | 133.04 | 126.55 | 131.28 | 254.15 | 210.27 | 144.50 | 138.45 | 83.15 | 0.00 | 219.16 |
| Benmore | 191.62 | 135.88 | 45.84 | 166.29 | 162.66 | 131.33 | 102.66 | 145.28 | 98.03 | 175.93 | 27.18 | 110.17 | 104.76 | 141.06 | 219.16 | 0.00 |

### **Table S5.7.** Geographic distance matrix at a maximum migration altitude of 2000 m

|  | Kaniere | Mapourika | Alexandrina | Grasmere | Pearson | Ellery | Clearwater | Ellesmere | Emma | Gunn | Middleton | Moeraki | Paringa | Selfe | Haupiri | Benmore |
| --- | --- | --- | --- | --- | --- | --- | --- | --- | --- | --- | --- | --- | --- | --- | --- | --- |
| Kaniere | 0.00 | 51.65 | 122.95 | 53.90 | 56.77 | 131.40 | 79.65 | 115.21 | 78.31 | 195.93 | 148.90 | 98.44 | 93.10 | 45.52 | 33.59 | 161.23 |
| Mapourika | 51.65 | 0.00 | 87.35 | 102.65 | 105.52 | 81.11 | 68.19 | 147.61 | 72.78 | 145.64 | 97.91 | 48.15 | 42.81 | 92.11 | 82.69 | 110.80 |
| Alexandrina | 122.95 | 87.35 | 0.00 | 127.88 | 125.01 | 126.58 | 67.29 | 116.47 | 63.51 | 181.50 | 46.58 | 97.51 | 92.17 | 106.58 | 154.43 | 39.46 |
| Grasmere | 53.90 | 102.65 | 127.88 | 0.00 | 3.18 | 182.40 | 79.61 | 64.91 | 75.39 | 246.93 | 159.75 | 149.44 | 144.10 | 28.91 | 47.47 | 144.28 |
| Pearson | 56.77 | 105.52 | 125.01 | 3.18 | 0.00 | 185.27 | 77.08 | 62.25 | 72.53 | 249.80 | 156.88 | 152.31 | 146.97 | 30.90 | 50.65 | 141.41 |
| Ellery | 131.40 | 81.11 | 126.58 | 182.40 | 185.27 | 0.00 | 149.28 | 227.85 | 153.87 | 64.57 | 89.37 | 33.87 | 39.41 | 172.41 | 162.44 | 111.22 |
| Clearwater | 79.65 | 68.19 | 67.29 | 79.61 | 77.08 | 149.28 | 0.00 | 79.99 | 4.59 | 213.81 | 102.08 | 116.32 | 110.98 | 54.32 | 112.07 | 87.49 |
| Ellesmere | 115.21 | 147.61 | 116.47 | 64.91 | 62.25 | 227.85 | 79.99 | 0.00 | 75.43 | 271.42 | 144.14 | 195.74 | 190.40 | 71.25 | 109.11 | 128.67 |
| Emma | 78.31 | 72.78 | 63.51 | 75.39 | 72.53 | 153.87 | 4.59 | 75.43 | 0.00 | 218.40 | 98.30 | 120.91 | 115.57 | 52.98 | 110.73 | 83.71 |
| Gunn | 195.93 | 145.64 | 181.50 | 246.93 | 249.80 | 64.57 | 213.81 | 271.42 | 218.40 | 0.00 | 141.21 | 98.40 | 103.94 | 236.94 | 226.97 | 150.88 |
| Middleton | 148.90 | 97.91 | 46.58 | 159.75 | 156.88 | 89.37 | 102.08 | 144.14 | 98.30 | 141.21 | 0.00 | 68.95 | 64.56 | 138.56 | 179.94 | 23.57 |
| Moeraki | 98.44 | 48.15 | 97.51 | 149.44 | 152.31 | 33.87 | 116.32 | 195.74 | 120.91 | 98.40 | 68.95 | 0.00 | 5.53 | 139.45 | 129.48 | 90.80 |
| Paringa | 93.10 | 42.81 | 92.17 | 144.10 | 146.97 | 39.41 | 110.98 | 190.40 | 115.57 | 103.94 | 64.56 | 5.53 | 0.00 | 134.11 | 124.14 | 86.41 |
| Selfe | 45.52 | 92.11 | 106.58 | 28.91 | 30.90 | 172.41 | 54.32 | 71.25 | 52.98 | 236.94 | 138.56 | 139.45 | 134.11 | 0.00 | 68.89 | 123.09 |
| Haupiri | 33.59 | 82.69 | 154.43 | 47.47 | 50.65 | 162.44 | 112.07 | 109.11 | 110.73 | 226.97 | 179.94 | 129.48 | 124.14 | 68.89 | 0.00 | 189.64 |
| Benmore | 161.23 | 110.80 | 39.46 | 144.28 | 141.41 | 111.22 | 87.49 | 128.67 | 83.71 | 150.88 | 23.57 | 90.80 | 86.41 | 123.09 | 189.64 | 0.00 |

### **Table S5.8.** Geographic distance matrix at a maximum migration altitude of 2200 m

|  | Kaniere | Mapourika | Alexandrina | Grasmere | Pearson | Ellery | Clearwater | Ellesmere | Emma | Gunn | Middleton | Moeraki | Paringa | Selfe | Haupiri | Benmore |
| --- | --- | --- | --- | --- | --- | --- | --- | --- | --- | --- | --- | --- | --- | --- | --- | --- |
| Kaniere | 0.00 | 46.77 | 103.00 | 45.51 | 47.95 | 119.23 | 68.10 | 101.43 | 67.19 | 177.02 | 129.05 | 89.23 | 84.45 | 38.98 | 30.12 | 137.44 |
| Mapourika | 46.77 | 0.00 | 70.70 | 89.66 | 92.10 | 73.57 | 57.64 | 128.96 | 61.63 | 131.36 | 82.73 | 43.57 | 38.79 | 78.06 | 74.83 | 92.35 |
| Alexandrina | 103.00 | 70.70 | 0.00 | 111.07 | 108.59 | 108.63 | 57.32 | 103.76 | 54.65 | 158.93 | 40.57 | 81.54 | 76.77 | 92.95 | 131.42 | 34.65 |
| Grasmere | 45.51 | 89.66 | 111.07 | 0.00 | 2.78 | 162.12 | 68.16 | 58.00 | 65.68 | 219.91 | 140.91 | 132.12 | 127.34 | 24.21 | 41.01 | 127.21 |
| Pearson | 47.95 | 92.10 | 108.59 | 2.78 | 0.00 | 164.56 | 67.17 | 55.61 | 63.20 | 222.35 | 138.43 | 134.56 | 129.78 | 25.22 | 43.79 | 124.73 |
| Ellery | 119.23 | 73.57 | 108.63 | 162.12 | 164.56 | 0.00 | 131.20 | 201.06 | 135.19 | 57.83 | 78.14 | 30.74 | 35.67 | 151.14 | 147.29 | 96.99 |
| Clearwater | 68.10 | 57.64 | 57.32 | 68.16 | 67.17 | 131.20 | 0.00 | 71.64 | 3.99 | 188.99 | 89.12 | 101.20 | 96.42 | 46.45 | 97.05 | 76.07 |
| Ellesmere | 101.43 | 128.96 | 103.76 | 58.00 | 55.61 | 201.06 | 71.64 | 0.00 | 67.68 | 241.83 | 129.24 | 172.52 | 167.74 | 63.69 | 96.00 | 115.54 |
| Emma | 67.19 | 61.63 | 54.65 | 65.68 | 63.20 | 135.19 | 3.99 | 67.68 | 0.00 | 192.98 | 86.11 | 105.19 | 100.41 | 45.22 | 96.14 | 73.06 |
| Gunn | 177.02 | 131.36 | 158.93 | 219.91 | 222.35 | 57.83 | 188.99 | 241.83 | 192.98 | 0.00 | 122.54 | 88.54 | 93.47 | 208.94 | 205.08 | 132.12 |
| Middleton | 129.05 | 82.73 | 40.57 | 140.91 | 138.43 | 78.14 | 89.12 | 129.24 | 86.11 | 122.54 | 0.00 | 58.75 | 55.08 | 122.92 | 157.11 | 20.81 |
| Moeraki | 89.23 | 43.57 | 81.54 | 132.12 | 134.56 | 30.74 | 101.20 | 172.52 | 105.19 | 88.54 | 58.75 | 0.00 | 4.93 | 121.14 | 117.29 | 77.60 |
| Paringa | 84.45 | 38.79 | 76.77 | 127.34 | 129.78 | 35.67 | 96.42 | 167.74 | 100.41 | 93.47 | 55.08 | 4.93 | 0.00 | 116.36 | 112.51 | 73.93 |
| Selfe | 38.98 | 78.06 | 92.95 | 24.21 | 25.22 | 151.14 | 46.45 | 63.69 | 45.22 | 208.94 | 122.92 | 121.14 | 116.36 | 0.00 | 58.77 | 109.22 |
| Haupiri | 30.12 | 74.83 | 131.42 | 41.01 | 43.79 | 147.29 | 97.05 | 96.00 | 96.14 | 205.08 | 157.11 | 117.29 | 112.51 | 58.77 | 0.00 | 165.86 |
| Benmore | 137.44 | 92.35 | 34.65 | 127.21 | 124.73 | 96.99 | 76.07 | 115.54 | 73.06 | 132.12 | 20.81 | 77.60 | 73.93 | 109.22 | 165.86 | 0.00 |

Species delimitation analysis using analysis of covariance

**Table S6.1.** Bootstrapped parameter estimates (1000 samples) of the analysis of covariance.

The table first shows the mean position of intercept, then deviation of each class of the group factor (“Within ABC clade comparisons”, “Within A. winterbourni clade comparisons” and “Between clade comparisons”, referring to pairwise distances within the ABC clade, within *A. winterbourni* clade and between them). The next parameter is the average slope (Ln-transformed geographic distance) followed by deviation of slopes of each group specific slope from the average. Column “Bias” illustrates the deviation between raw estimates and bootstrapped estimates, followed by bootstrapped significance and confidence interval values.

| Parameter | B | Bootstrap | | | | |
| --- | --- | --- | --- | --- | --- | --- |
|  |  | Bias | Std. Error | Sig. (2-tailed) | 95% Confidence Interval | |
|  |  |  |  |  | Lower | Upper |
| Intercept | -4.468 | -0.005 | 0.324 | 0.001 | -5.093 | -3.828 |
| Within ABC clade comparisons | -1.433 | 0.022 | 1.012 | 0.146 | -3.299 | 0.657 |
| Between clade comparisons | 2.544 | -0.002 | 0.454 | 0.001 | 1.605 | 3.436 |
| Within A. winterbourni clade comparisons | 0 | 0 | 0 |  | 0 | 0 |
| Ln-transformed geographic distance | 0.166 | 0.001 | 0.054 | 0.003 | 0.059 | 0.269 |
| Within ABC clade comparisons * geographic distance | 0.249 | -0.004 | 0.170 | 0.132 | -0.108 | 0.554 |
| Between clade comparisons * geographic distance | -0.267 | 0.001 | 0.076 | 0.001 | -0.414 | -0.110 |
| Within A. winterbourni clade * geographic distance | 0 | 0 | 0 |  | 0 | 0 |

**Table S6.2.** Bootstrapped parameter estimates for the ABC clade.

Bootstrapped estimates are based on 956 samples.

| Parameter | B | Bootstrap | | | | |
| --- | --- | --- | --- | --- | --- | --- |
|  |  | Bias | Std. Error | Sig. (2-tailed) | 95% Confidence Interval | |
|  |  |  |  |  | Lower | Upper |
| Intercept | -5.042 | 0.008 | 0.701 | 0.001 | -6.305 | -3.601 |
| Group C versus groups A & B | 0.548 | -0.005 | 0.079 | 0.001 | 0.389 | 0.710 |
| Within groups A & B | 0.353 | -0.006 | 0.131 | 0.007 | 0.061 | 0.562 |
| Within group C | 0 | 0 | 0 |  | 0 | 0 |
| Ln-transformed geographic distance | 0.223 | -0.001 | 0.119 | 0.068 | -0.020 | 0.446 |

**Table S6.3**. Bootstrapped parameter estimates for *A. winterbourni* clade.

Bootstrapped estimates are based on 995 samples.

| Parameter | B | Bootstrap | | | | |
| --- | --- | --- | --- | --- | --- | --- |
|  |  | Bias | Std. Error | Sig. (2-tailed) | 95% Confidence Interval | |
|  |  |  |  |  | Lower | Upper |
| Intercept | -4.400 | 0.006 | 0.338 | 0.001 | -5.060 | -3.722 |
| Groups E & M versus groups W & CA | -0.025 | -0.001 | 0.040 | 0.532 | -0.104 | 0.051 |
| Within groups E & M | -0.153 | -0.001 | 0.077 | 0.035 | -0.321 | -0.017 |
| Within groups W & CA | 0 | 0 | 0 |  | 0 | 0 |
| Ln-transformed geographic distance | 0.158 | -0.001 | 0.057 | 0.006 | 0.044 | 0.272 |

References

Bankers, L., & Neiman, M. (2017). De Novo Transcriptome Characterization of a Sterilizing Trematode Parasite (*Microphallus* sp.) from Two Species of New Zealand Snails. *G3 (Bethesda, Md.), 7*(3), 871-880. doi:10.1534/g3.116.037275

Blasco-Costa, I., Seppala, K., Feijen, F., Zajac, N., Klappert, K., & Jokela, J. (2019). A new species of *Atriophallophorus* Deblock & Rose, 1964 (Trematoda: Microphallidae) described from in vitro-grown adults and metacercariae from *Potamopyrgus antipodarum* (Gray, 1843) (Mollusca: Tateidae). *Journal of Helminthology, 94*, e108. doi:10.1017/S0022149X19000993

Excoffier, L., & Lischer, H. E. (2010). Arlequin suite ver 3.5: a new series of programs to perform population genetics analyses under Linux and Windows. *Molecular ecology resources, 10*(3), 564-567. doi:10.1111/j.1755-0998.2010.02847.x

Feijen, F. A. A. (2020). *Looking-Glass Parasites.* (PhD), ETH Zürich, Switzerland, Zürich. (Diss ETH 26942 )

Kitada, S., Nakamichi, R., & Kishino, H. (2017). The empirical Bayes estimators of fine-scale population structure in high gene flow species. *Molecular ecology resources, 17*(6), 1210-1222. doi:10.1111/1755-0998.12663

Lively, C. M., & McKenzie, J. C. (1991). Experimental infection of a freshwater snail, *Potamopyrgus antipodarum*, with a digenetic trematode, *Microphallus* sp. *New Zealand Natural Sciences, 18*, 59-62. doi:10.26021/340

Osnas, E. E., & Lively, C. M. (2011). Using definitive host faeces to infect experimental intermediate host populations: waterfowl hosts for New Zealand trematodes. *New Zealand Journal of Zoology, 38*(1), 83-90. doi:10.1080/03014223.2010.528779

Paradis, E. (2010). pegas: an R package for population genetics with an integrated-modular approach. *Bioinformatics, 26*(3), 419-420. doi:10.1093/bioinformatics/btp696

Pérez-Ponce de León, G., & Hernández-Mena, D. I. (2019). Testing the higher-level phylogenetic classification of Digenea (Platyhelminthes, Trematoda) based on nuclear rDNA sequences before entering the age of the 'next-generation' Tree of Life. *J. Helminthol., 93*(3), 260-276. doi:10.1017/S0022149X19000191

Ryman, N., & Palm, S. (2006). POWSIM: a computer program for assessing statistical power when testing for genetic differentiation. *Molecular Ecology*(6), 600-602. doi:10.1111/j.1365-294X.2006.01378.x

Wang, I. J. (2020). Topographic path analysis for modelling dispersal and functional connectivity: Calculating topographic distances using the topoDistance r package. *Methods in Ecology and Evolution, 11*(2), 265-272. doi:10.1111/2041-210X.13317
